# The quorum-sensing regulator ComA from *Bacillus subtilis* activates transcription using topologically distinct DNA motifs

**DOI:** 10.1093/nar/gkv1242

**Published:** 2015-11-17

**Authors:** Diana Wolf, Valentina Rippa, Juan Carlos Mobarec, Patricia Sauer, Lorenz Adlung, Peter Kolb, Ilka B. Bischofs

**Affiliations:** 1 Center for Molecular Biology (ZMBH) and Center for the Quantitative Analysis of Molecular and Cellular Biosystems (BioQuant), University of Heidelberg, Im Neuenheimer Feld 267, 69120 Heidelberg, Germany; 2 Department of Pharmaceutical Chemistry, Philipps-University Marburg, Marbacher Weg 6, 35032 Marburg, Germany

## Abstract

ComA-like transcription factors regulate the quorum response in numerous Gram-positive bacteria. ComA proteins belong to the tetrahelical helix-turn-helix superfamily of transcriptional activators, which bind as homodimers to inverted sequence repeats in the DNA. Here, we report that ComA from *Bacillus subtilis* recognizes a topologically distinct motif, in which the binding elements form a direct repeat. We provide *in vitro* and *in vivo* evidence that the canonical and non-canonical site play an important role in facilitating type I and type II promoter activation, respectively, by interacting with different subunits of RNA polymerase. We furthermore show that there is a variety of contexts in which the non-canonical site can occur and identify new direct target genes that are located within the integrative and conjugative element ICEBs1. We therefore suggest that ComA acts as a multifunctional transcriptional activator and provides a striking example for complexity in protein–DNA interactions that evolved in the context of quorum sensing.

## INTRODUCTION

Most bacterial species are confronted with frequent and often extreme changes in environmental conditions, to which they must rapidly adapt. To this end, they monitor many aspects of their environment using receptor-based signaling systems which, when triggered, alter their phenotypic properties. One major class of regulatory network is involved in ‘quorum sensing’ control, which is defined as the regulation of gene expression in response to cell density. In many bacteria, quorum sensing brings about global changes in gene expression and is commonly involved in regulating complex physiological behaviors (1). These traits typically confer an adaptive advantage on the bacteria in crowded or otherwise constricted surroundings, enabling them to survive in competitive environments and/or adapt to and interact with the environment of a host. Quorum sensing is a common regulatory strategy in microbes, and has apparently evolved several times in parallel. Hence, there exists a diversity in overall quorum-sensing network designs that extends to the molecular level (2,3). For instance, the master regulators of quorum sensing in *Vibrio* species, including LitR (*V. fischeri*), HapR (*V. cholerae*) and LuxR (*V. harveyi*), belong to the TetR family of transcriptional repressors (4). In contrast, both TraR in *Agrobacterium tumefaciens* (5) and ComA in *Bacillus subtilis* (6) belong to the GerE/NarL/LuxR (*V. fischeri*) family of transcriptional activators, while others, such as members of the Rgg family in *Streptococcus pyogenes* (7) belong to the RNPP-family (8–10).

Despite this apparent diversity, different quorum sensing systems possess certain common features. Among these is a tendency for the transcription factors that mediate the quorum response to evolve the ability to engage in more complex types of gene regulation. For example, LuxR (*V. harveyi*) (11,12) and its homologue HapR (*V. cholerae*) (13) can act both as TetR-like repressors and as activators, respectively. Moreover, the functional versatility exhibited by these transcription factors is reflected in altered interactions of the response regulator with the DNA. While the LuxR homologues bind to repressor sites by interacting with DNA via a canonical TetR-like binding site exhibiting dyad symmetry, the activator site they recognize resembles the canonical site but has lost its structural symmetry (12,13). The ability of these transcription factors to bind DNA and regulate transcription in alternative ways enables differential control over a large set of target genes.

In one of the best studied Gram-positive model organisms, the bacterium *Bacillus subtilis*, more than 10% of the genome is controlled by a sophisticated quorum sensing network that is centered on the transcription factor ComA (14). In response to various signaling peptides, ComA controls many important physiological behaviors in this organism (15–18) and—since close homologues of ComA and upstream signaling genes have been identified in other *Firmicutes* (19)—most probably in many other bacteria also. Structurally, ComA belongs to the NarL family of transcriptional activators. Its C-terminal DNA-binding domain shares sequence homology with LuxR, the regulator of the quorum response in *V. fischeri*, and is a member of the widespread tetrahelical helix-turn-helix (HTH) subfamily that accounts for about 25% of all DNA-binding response regulators in bacteria (20,21). The basic HTH domain is required for DNA binding, while the additional fourth helix provides a dimerization interface that facilitates formation of C-terminal face-to-face domain dimers (6). Moreover, the mirror symmetry of the domain dimer is reflected in the architecture of the canonical DNA binding site, which displays dyad symmetry (22). Each protomer binds to one of the two recognition elements, which are separated in the DNA by a spacer segment that bridges the dimer interface. As demonstrated by *in vivo* (14,23–24)*, in vitro* (25) and structural studies (6), ComA binds to a canonical binding site that can be identified as an inverted repeat (IR) of a specific recognition element in the DNA. However, a recent study has suggested that ComA may interact with DNA in more complex ways, since ComA target promoters contain an additional, relatively degenerate recognition element that is located downstream of the canonical binding site. This putative ‘half-site’ has been shown to have important functions in the control of transcription *in vivo* (24). Nevertheless its existence is puzzling, as ComA is a dimer in solution and interacts with DNA as a homodimer (6,24,26).

Here, we report the discovery of a second, non-canonical DNA binding site for ComA. This binding sequence has a direct-repeat (DR) configuration, and we show that it displays properties quite different from those of the canonical IR. We elucidate this atypical form of transcription regulation by a quorum sensing regulator and provide *in vitro* and *in vivo* experimental evidence that the identity of the binding sites influences the interactions of the transcription factor with the transcriptional machinery. We identify new target genes of ComA and reveal a surprising degree of complexity and diversity in the architecture and function of ComA-regulated target promoters. We therefore suggest that ComA has acquired the ability to interact with DNA in more complex ways, and functions as a multifunctional transcriptional activator. Our results, together with recent findings relating to the TetR-like LuxR homologues in *Vibrio* species, further underline an emerging theme: quorum sensing control systems exhibit characteristics that appear to promote molecular innovation in protein–DNA interactions over the course of evolution.

## MATERIALS AND METHODS

### Bacterial strains and media

*Escherichia coli* DH5α (Invitrogen, Carlsbad, CA, USA) was used for cloning. All *B. subtilis* strains were derived from either 1A700 (W168) or JH642 (see Supplementary Table S1). Strains were grown in Luria–Bertani broth (LB), S7_50_ minimal medium (27) or Difco sporulation medium (DSM) (28) at 37°C with aeration. LB agar plates were used to select transformants. When required, the appropriate antibiotics and amino acids were added as follows – for *E. coli:* ampicillin (100 μg ml^−1^), kanamycin (50 μg ml^−1^); for *B. subtilis:* chloramphenicol (5 μg ml^−1^), kanamycin (10 μg ml^−1^), spectinomycin (100 μg ml^−1^), erythromycin (1 μg ml^−1^) plus lincomycin (25 μg ml^−1^) for MLS selection, tryptophan (50 μg ml^−1^) and phenylalanine (50 μg ml^−1^), X-Gal (100 μg ml^−1^).

### Plasmid construction

All plasmids and primers are listed in Supplementary Tables S2 and S3, respectively.

For production of recombinant ComA, α and σ^A^ as N-terminal His_6_ fusion proteins in *E.coli*, the corresponding genes were amplified from the bacterial chromosome of *B. subtilis* W168 and cloned into the expression vector pBAD18 (29) following standard procedures.

Reporter constructs used to assess promoter activity were assembled by ligation-independent cloning (LIC) (30) into the plasmid pYFPbglS (31). Wild-type promoter constructs (P*_srfAA_*, P*_rapC_*, P*_rapA_*, P*_pel_*, P*_lutP_*, P*_ybaJ_, P_ylbO_, P_ykhA_*, P*_yddk_* and P*_rapI_*) were amplified by PCR from the bacterial chromosome of *B. subtilis* W168 using the primers indicated in Supplementary Table S3. Mutants of P*_rapA_*, P*_lutP_* and P*_rapC_* were created by PCR using primers with appropriate nucleotide substitutions in the indicated recognition elements. To introduce mutations into P*_srfAA_* up- and downstream promoter fragments were amplified from plasmid pDW40 using primers carrying mutations in the indicated recognition element(s). Subsequently, the fragments were fused by overlap-extension PCR and cloned into pYFPbglS. Synthetic promoters containing combinations of perfect direct repeats (PDR) and perfect inverted repeats (PIR) were constructed analogously. gDNA fragments of P*_srfAA_* were amplified with primer pairs DW76/ST11 and DW78/ST1 to mutate the native IR and DR sites to the idealized motifs. In a second PCR, the resulting PCR fragments were joined with appropriate primer pairs DW77/ST11 and DW79/ST11 to construct PIR-PIR or PIR-PDR and PDR-PDR or PDR-PIR promoters, respectively. Fused PCR products were cloned into pGFPbglS (plasmids pDW54–57). These plasmids then served as templates for constructing the respective iYFP fusions in pYFPbglS (plasmids pDW97–100).

To construct a clean deletion of *comA*, we amplified 1-kb fragments flanking the *comA* gene from the bacterial chromosome. Up- and downstream fragments were fused by overlap-extension PCR and cloned into pMAD (32) with BamHI and EcoRI, resulting in plasmid pDW6.

All constructs were verified by sequencing across the inserts using appropriate primers.

### Strain construction

Fluorescent reporter strains were obtained by transforming *B. subtilis* with the indicated YFP reporter plasmids according to standard protocols (28). Correct integration of the constructs at the *bglS* locus was verified by PCR.

Promoters of putative ComA target genes were also tested in reporter constructs introduced into a *comA* deletion mutant. As the *comA* deletion strain has lost natural competence, we used BIB372, which carries the replicative plasmid pLK (33), as the host strain. This plasmid allows us to induce expression of the master regulator of competence ComK, thereby ensuring that the *comA* deletion strain is amendable to further genetic manipulations. The *comA* mutant was constructed using plasmid pDW6 following a protocol similar to that of Arnaud *et al*. (32). The resulting strain BIB396, carrying a *comA* gene deletion, was verified by PCR and sequencing. BIB396 was transformed with chromosomal DNA from fluorescent reporter strains and subsequently cured of the pLK plasmid as described by Nijland *et al*. (33). Correct integration of the fluorescent reporter into the *bgl* locus and absence of *comA* in the reporter strains were verified by appropriate PCRs. The resulting strains are listed in Supplementary Table S1.

### Electrophoretic mobility shift assays (EMSAs)

His_6_-tagged ComA protein, the α−subunit of RNA polymerase (RNAP) and the sigma-factor σ^A^ were purified on Ni-NTA columns (Macherey Nagel). ComA was purified as described before (24) and α and σ^A^ were purified using the standard protocol provided by the manufacturer. All electrophoretic mobility shift assay (EMSA) probes are listed in Supplementary Table S3. Annealing was performed by incubating sense and antisense oligonucleotides at 95°C for 5 min and allowing the mixture to cool gradually to room temperature. DNA probes containing entire promoters were PCR-amplified from genomic DNA using the indicated primers (Supplementary Table S3). EMSA reactions (15 μl) contained 10 ng of biotinylated double-stranded DNA and the indicated amounts of recombinant ComA, α and σ^A^ dissolved in 1X binding buffer (10 mM HEPES pH 7.6, 2 mM MgCl_2_ × 6 H_2_O, 0.1 mM EDTA, 200 mM KCl, 5 mM DTT, 10% glycerol). Reactions were run for 20 min at room temperature. Protein–DNA complexes were separated on a native 10% (synthetic constructs) or 8% (wild-type promoters) polyacrylamide gel in 0.5x TBE buffer (45 mM Tris pH 8.0, 45 mM boric acid, 1 mM EDTA) at 80 V (20 V/cm). Electrophoretic transfer of DNA to a nylon membrane was carried out in 0.5x TBE buffer at 380 mA for 1 h and DNA was cross-linked to the membrane with UV light. After incubation (1 h) in blocking buffer, the membrane was incubated for 1 h with streptavidin–HRP conjugate (Thermo Scientific). The membrane was washed and visualized with the SuperSignal chemiluminescent reagent (Pierce). DNA fragments corresponding to PDR mutants into which single transition mutations had been introduced at positions 1, 3, 5 of RE3 and at positions 5, 6 of RE4 were amplified by PCR using the non-biotinylated oligonucleotides listed in Supplementary Table S3. In this case, DNA bands were visualized with SYBR Safe (Invitrogen) after electrophoresis.

To obtain a ComA binding curve, quantitative EMSA titrations were performed. Quantification of free DNA and protein-bound DNA was performed by densitometry using ImageJ software (NIH). All DNA-binding experiments were performed in triplicates. The average percentage of bound DNA was plotted versus the protein concentration. Curve fitting, determination of dissociation constant and Hill coefficient were performed using GraphPad Prism 5 software.

### Fluorescence reporter assays

*B. subtilis* strains were inoculated into 3-ml tubes of LB containing the appropriate antibiotics, and shaken at 180 rpm for 6 h at 37°C, then resuspended in S7_50_ medium (w/o antibiotics) to a starting optical density at 600 nm (OD_600_) of 0.02 and grown overnight (16 h) at 30°C. Samples were diluted into 10 ml of fresh S7_50_ medium (OD = 0.02) and grown at 37°C with vigorous aeration to OD = 3, at which point the activity of the respective promoters was assayed by single-cell fluorescence microscopy. To test for new potential ComA target genes, the promoter activity was measured at various ODs. For measurements on the *rapI* promoter, cells were grown in DSM (28). Samples for microscopy were prepared as described before (34) with the exception that the agarose pads contained phosphate buffered saline (PBS) (137 mM NaCl, 2.7 mM KCl, 10 mM Na_2_HPO_4_, 2 mM KH_2_PO_4_). Fluorescence and bright-field images were taken on a DeltaVision Elite Imaging System (Applied Precision, Issaquah, WA, USA) equipped with a solid-state light source, a sCMOS PCO Edge camera and a UPlanSApo 100x/1.40 na oil objective (Olympus, Tokyo, Japan). Cells were exposed for 0.5 s at 100% intensity using the YFP filter set (513 / 17 nm, 559 / 38 nm). Images were binned 2 × 2 using SoftWoRx 5.5 software. Bright-field images were segmented using a customized software (35) and manually inspected. For each cell, the mean fluorescence intensity was determined from the segmented area and the background fluorescence was subtracted. Between 500 and 1000 cells were analyzed per condition and experiment. For all strains, results are based on three independent experiments, each performed on cells from two separate clones, and the data are presented as means ± standard deviation. *P* values were calculated using two-tailed Student's *t-*test. An asterisk indicates a *P* value of <0.05, two asterisks indicate a *P* value of <0.01, three asterisks indicate a *P* value of <0.001 and ns (not significant) corresponds to a *P* value of >0.05.

## RESULTS

### Sequence alignments provide evidence for an alternative binding motif for ComA

**Figure 1.**
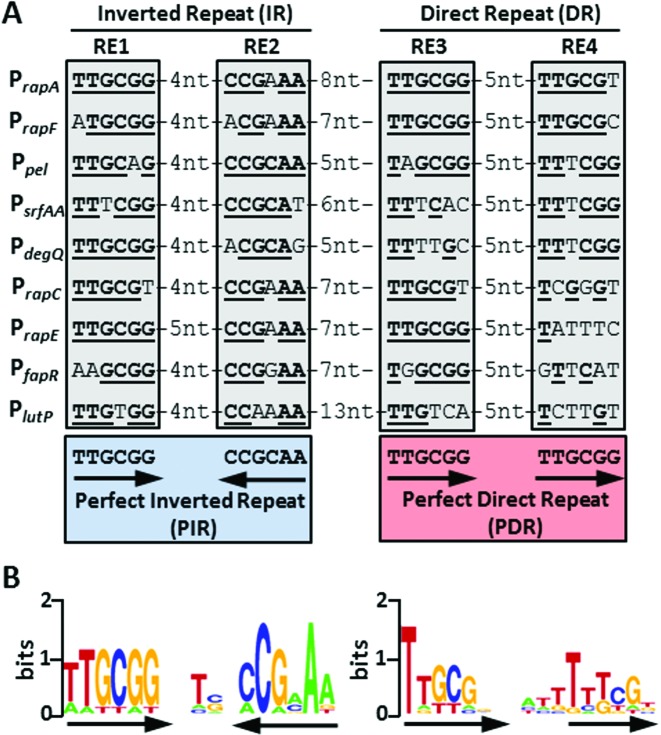
Sequence-based evidence for a novel ComA DNA-binding motif. (**A**) Sequence alignment of all known ComA-regulated target promoters. The grey boxes denote the hexameric recognition elements. Two such REs, RE1 and RE2, are arranged as an inverted repeat (IR) with dyad symmetry and make up the canonical ComA binding site (blue box). Two additional putative REs, RE3 and RE4, are oriented as a direct repeat (DR) with a spacer, that has a highly conserved length of 5 nt (red box). The underlined, bold letters in each RE indicate a match with respect to the idealized symmetric sequence motif shown at the bottom. (**B**) ComA DNA-binding motifs of the IR and DR units derived from MEME analysis of the sequences shown in A. Arrows indicate the symmetry properties of the respective sequence blocks.

Although ComA mediates global changes in gene expression in *B. subtilis*, only about 20 genes, organized in 9 transcriptional units, are implied to be under its direct control (14). The promoter sequences of these genes each contain at least one canonical binding site upstream of the RNAP binding site. This element comprises a classical IR made up of two hexameric recognition elements (REs) with the consensus sequence TTGCGG separated by a 4-nucleotide spacer (23,25) (Figure 1A). The corresponding DNA motif derived from MEME analyses (36) of the indicated sequences in Figure 1A is shown in Figure 1B (left). This shows clear evidence of dyad symmetry and the idealized symmetrical motif is a PIR. Interestingly, upon aligning the promoter sequences we noticed a second, related repeat nearby. This shares the same hexameric recognition element with the canonical binding site, but here they are arranged as a direct repeat with a 5-nt spacer between them. The idealized motif may be described by a PDR given by TTGCGG-5nt-TTGCGG. Clear signatures of this motif can be found in most known target promoters of ComA in *B. subtilis* a short distance downstream of the canonical site (Figure 1A). In Figure 1B (right) we show the sequence logo computed from the short sequence elements present in the nine known ComA target promoters. The DR motif is considerably more degenerate than the IR, with the element closer to the canonical binding site being more highly conserved. This part of the DR motif corresponds to the ‘half-site’ (RE3) previously identified and studied (24). The relative degeneracy of the second ‘half-site’ (RE4) obscures the symmetry properties in the resulting sequence logo. Nevertheless, some promoters have a very well conserved DR. Thus, P*_rapA_* and P*_rapF_* each differ from the idealized motif by only a single mismatch. In other promoters, RE3 or RE4 (e.g. P*_rapC_* or P*_srfAA_*) or both (e.g. P*_lutP_*) may deviate from the idealized symmetric sequence by more than 50%.

### The direct repeat serves as a functional regulatory element in ComA-dependent promoters

**Figure 2.**
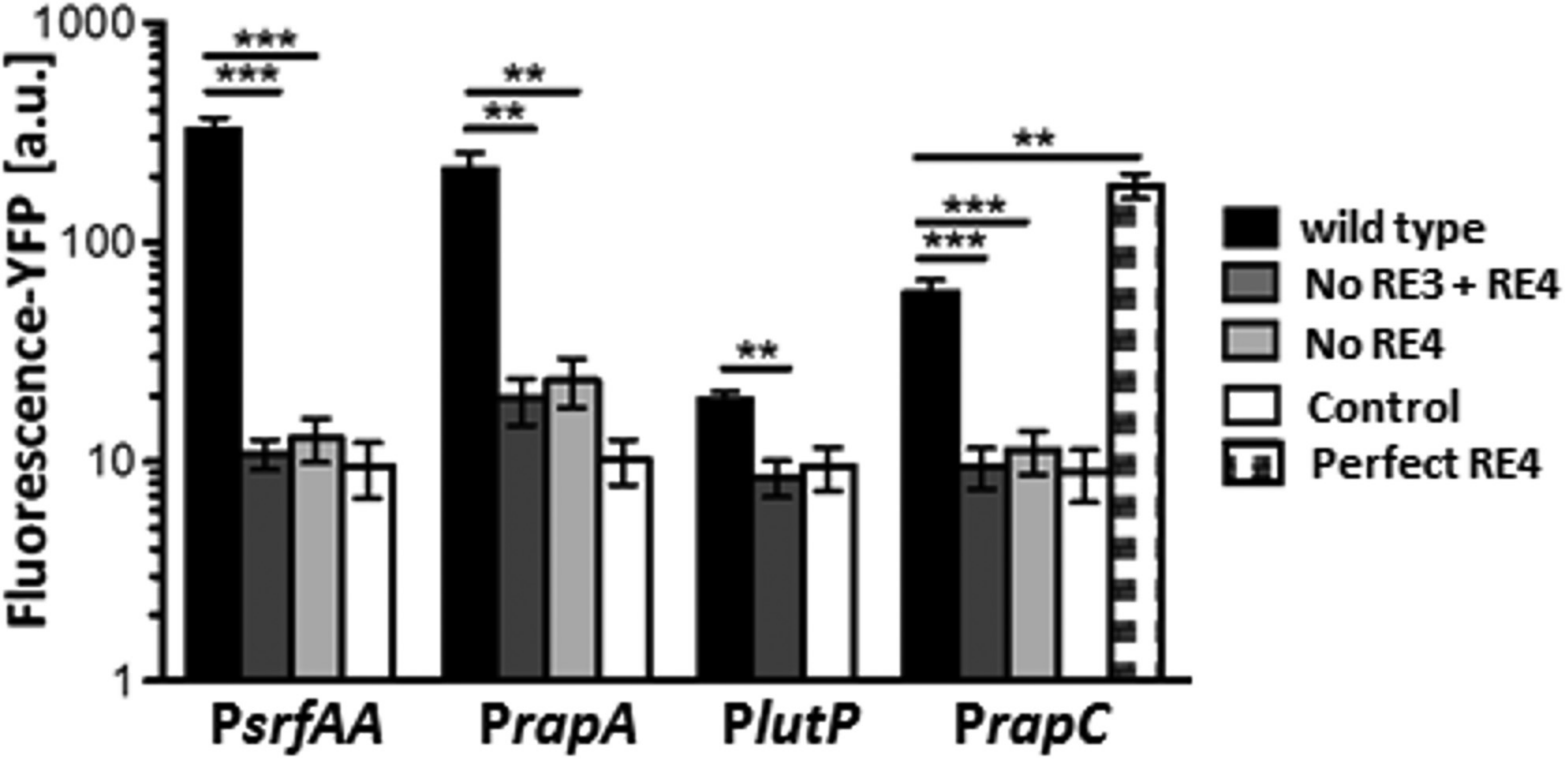
The DR is a novel functional element of ComA-regulated promoters. The DR contributes to the activation of four ComA target promoters. Activities of the indicated wild-type promoters (black bars) were compared to those of mutant promoters containing nucleotide substitutions in the indicated REs (grey and dotted bars) based on expression levels of the YFP reporter. A strain carrying a promoter-less *iyfp* was used as control (white bars). Mean cellular fluorescence was determined from the fluorescence distribution of at least 500 cells assayed by quantitative fluorescence microscopy. Error bars indicate the standard deviation calculated for three independent experiments involving two separate clones each. Mutations in REs comprising the DR that increase divergence from the idealized sequence (grey bars) reduce promoter activity, while mutations toward the idealized RE4 in P*rapC* (dotted bar) result in increased activity. Two asterisks indicate a *P* value of <0.01 and three asterisks indicate a *P* value of <0.001.

To test whether the presence of a DR element in the promoter affects transcription *in vivo*, mutant promoters fused to a YFP reporter protein were ectopically integrated in single copy into the chromosome of *B. subtilis* 168. We chose promoters exhibiting various degrees of conservation in their respective DR elements (*rapA, rapC, srfAA* and *lutP*) and mutated the DR sequence in their respective promoters (Supplementary Table S4). We then compared the activity of each mutant promoter to that of its wild-type (*wt*) counterpart using quantitative single-cell fluorescence microscopy to assay the mean cellular fluorescence intensity. A strain carrying a promoterless *iyfp* reporter gene was used as a control. In the mutant strains carrying no DR element, the fluorescence dropped to levels comparable to that of the promoterless control. In the case of *rapA*, activity was strongly reduced in the absence of the DR element, but low residual activity was still detectable (Figure 2). Since the DR contains the half-site (RE3) known to be important for promoter activation *in vivo* (24), we next focused on the contribution of the second half-site (RE4). We replaced RE4 in the *rapA, rapC* and *srfAA* promoters, respectively, by a mutated sequence. In all cases, fluorescence intensity again fell to values that were not significantly different from those for the same promoters lacking the entire DR. To further explore the functional significance of the RE4, we then substituted the idealized sequence of the DR for the fairly degenerate RE4 element in the *rapC* promoter. If the element plays a role in ComA-dependent transcription *in vivo*, this mutation might increase its affinity for ComA and thus lead to an increase in promoter activity. Indeed, as expected, the alteration enhanced transcriptional activity, resulting in a 3-fold increase in fluorescence intensity. Analogous observations were made when the degenerate RE3 in the *srfAA* promoter was mutated toward or away from the consensus (24). Taken together, these results suggest that both RE3 and the newly identified RE4 comprising the DR contribute to transcription activation *in vivo*.

### DR and IR motifs define distinct binding site units for ComA

**Figure 3.**
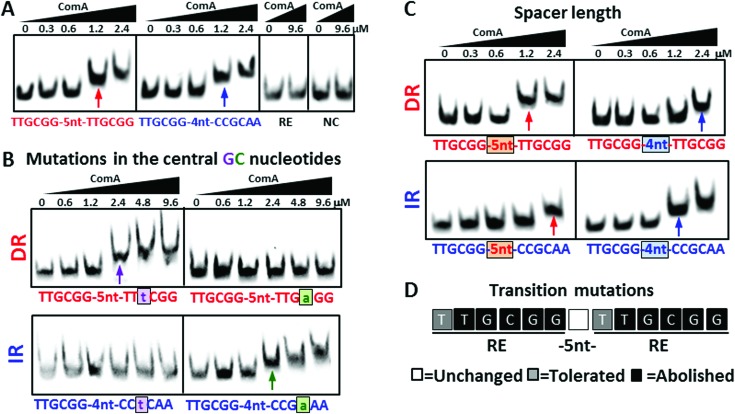
The DR and the IR define distinct ComA binding site units. (**A**) EMSAs in which 50 bp DNA probes (PDR = perfect direct repeat, PIR = perfect inverted repeat, RE = single recognition element, NC = negative control) were incubated with the indicated amounts of ComA. The lower bands represent free DNA. The upper bands (arrows) represent ComA–DNA complexes formed with PDR and PIR at ComA concentrations of 1.2 μM or more. No complexes formed with a single RE up to 9.6 μM. The data are representative of three independent experiments each. (**B**) Results from EMSAs of PDR (top) and PIR (bottom) probes carrying identical substitutions in the central CG nucleotides. Left: G3 → T, Right: C4 → A. The substitutions differentially affect ComA-binding to IR and DR sequences. (**C**) Results from EMSAs for PDR-variants (top) and PIR-variants (bottom) by swapping the spacer length n between the REs. DR configurations have the highest affinity for n = 5 (left) while for IR configurations, n = 4 (right) is preferred. (**D**) Effects of single base substitutions within the PDR on ComA binding. The sequence of the PDR is shown and the effects of transition mutations in EMSAs are indicated by the color coding of the boxes. White box = no change in binding affinity; grey box = 2-fold reduced binding affinity; black box = no ComA binding at 9.6 μM. All point mutations reduced the affinity for ComA and in most cases the reduction was severe.

Interestingly, previous DNAase experiments on the *srfAA* promoter showed that ComA protected DNA regions that coincide with canonical IR-type binding sites and, in addition, a region close to the -35 region that overlaps with the proposed DR element (25). This suggests that the regulatory function of the DR-motif on promoter activation is executed by ComA. Hence, ComA could recognize both, IR and DR motifs. In order to test whether ComA is indeed able to bind to each motif, we performed EMSAs with recombinant His_6_-tagged ComA using short 50-bp DNA fragments with concentrations of ComA up to 9.6 μM. These probes contained at their center either an idealized canonical sequence (i.e. the PIR), an idealized non-canonical sequence (the PDR), an idealized single RE or a negative control fragment (NC), in which the positions of the nucleotides comprising the REs were randomized. We found that ComA binds to fragments containing the canonical or non-canonical sequence elements *in vitro* with similar affinities. Moreover, the resulting molecular complexes show the same mobility. We also verified that single REs do not support ComA binding. We did not observe any binding to a single RE even at an 8-fold higher concentration than what is required for binding to PIR and PDR motifs, respectively (Figure 3A). These findings demonstrate that ComA can bind to quite different sequence motifs.

We next asked whether the proposed PIR and PDR motifs truly define prototype sequences for two distinct ComA binding sites. Note that in both sequence logos, the first half-site (i.e. RE1 and RE3, respectively) is more conserved than the second half-site (Figure 1B). Moreover, both motifs share two nucleotides GC at position three and four in their respective second half-site (i.e. RE2 = CC**GC**AA and RE4 = TT**GC**GG). It is therefore conceivable that ComA prefers to bind to a single (potentially asymmetric) IR-type motif and hence all IR and putative DR sites might be derivatives from a common motif. On the other hand, if in sequence space PIR and PDR sequences give rise to two local minima in the free energy landscape for ComA binding, any local deviation from the respective idealized motifs should decrease the affinity for ComA. We therefore investigated how sequence variations around the respective proposed idealized PDR and PIR motifs affect ComA binding in each case.

Firstly, we focused on the shared nucleotides (GC) in the central position of second RE. We specifically asked whether mutations existed that would distinguish the two sites. Based on the information content provided by the two sequence logos (Figure 1B), we reasoned that mutating G to T might abolish ComA binding in an IR-context but the identical mutation might be tolerated in a DR-context. Conversely, mutating C to A might abolish ComA binding in a DR-context but not in an IR-context. EMSA experiments on the respective mutant DNAs show that this is indeed the case (Figure 3B). ComA can bind to DR (G→T) and IR (C→A) albeit with an approximately 2-fold reduced affinity in each case. On the other hand, ComA does not bind to DR (C→A) and IR (G→T) up to concentrations of 9.6 μM. Hence, PIR and PDR sites respond differentially to identical perturbations in the central nucleotides.

Secondly, we studied the effect of the spacer length *n* between the two REs comprising the PDR and the PIR, respectively (Figure 3C). For a DR configuration we found that *n =* 5 is optimal for binding, although binding can still occur at higher concentrations of ComA if *n =* 4. All other spacer lengths from *n = 1–10*, including the addition of a complete turn of the DNA to the optimal spacing (*n = 15*) abolished binding in the tested concentration regime (data not shown). In contrast, for an IR configuration *n* = 4 is optimal for binding, while *n* = 5 is tolerated and all other lengths (from *n = 1–10* as well as *n = 14*) are again incompatible with binding. Thus, IR and DR have different optimal spacer lengths. These match to the proposed PIR and PDR motifs. Moreover, all DRs in known ComA target promoters (Figure 1A) have the predicted optimal DR-spacer. For IRs again the predicted optimal spacer is present in all promoter binding sites except for P*_rapE_*, which has the sub-optimal *n* = 5 spacer. These findings further support the idea that both motifs represent distinct entities.

Finally, we introduced single transition mutations at each position in the PDR (Figure 3D). All such point-mutations (outside the spacer region) in either RE affected binding severely – and in identical ways. In fact, only mutations in the first position of each RE were tolerated, reducing binding affinity by approximately 2-fold while all others abolished binding in the tested concentration regime and hence require at least a more than 8-fold increase of ComA for binding. Hence, every perturbation to the proposed idealized PDR motif diminished the ability to bind ComA. Moreover, the extent to which the affinity is reduced for the vast majority of point-mutations studied here hints at surprisingly stringent sequence requirements for ComA to bind to a single DR element.

Taken together, our binding experiments strongly suggest that there exist two alternative DNA binding site units for ComA which can be differentiated based on the topological properties of the underlying sequence motif. One is the canonical IR and the other is a non-canonical binding site motif in form of a DR.

### ComA functions in type I and type II promoter activation by interacting with different subunits of RNAP

**Figure 4.**
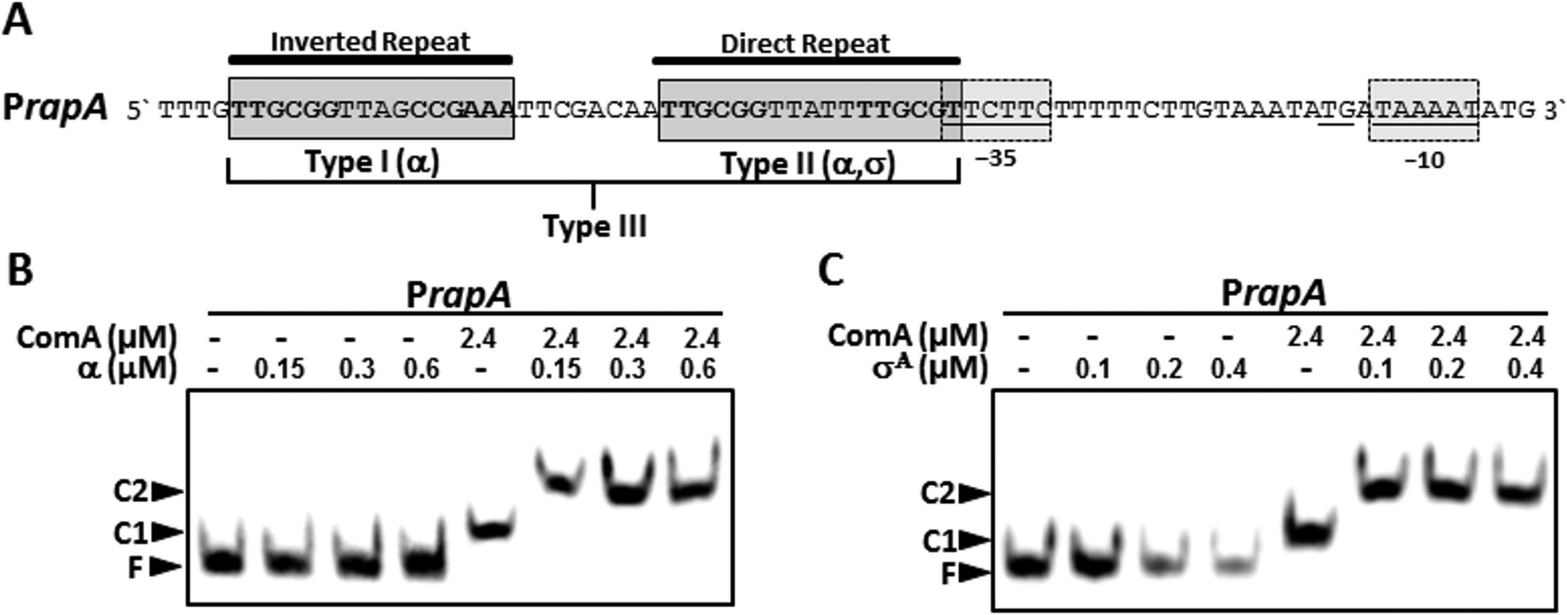
ComA interacts with different RNAP subunits at the *rapA* promoter. (**A**) Organization of the *rapA* promoter. The -35 and -10 hexamers for RNAP binding are underlined and enclosed in dashed boxes. The extended -10 element containing the dinucleotide TG is underlined. The positioning of the IR and DR-sequences (dark grey boxes) relative to the -35 element is indicative of a class I and class II activation by ComA, respectively, suggesting that P*_rapA_* is a class III promoter. (**B**) EMSAs of the *rapA* promoter with ComA and the α-subunit of RNAP. F: free DNA, C1: ComA-bound to P*_rapA_* (full occupancy), C2: ternary complex with the α-subunit. (**C**) EMSAs of the *rapA* promoter with ComA and σ^A^. F: free DNA, C1: ComA-bound to P*_rapA_* (full occupancy), C2: ternary complex with σ^A^.

In order to gain insight into the function of the two alternative binding sites in regulating transcription, we analyzed the structure of the promoter region of ComA-regulated genes in more detail. In many target promoters, the IR and DR together describe a regulatory core module that is located close to a poorly conserved -35 region and a -10 region with an extended sigma motif, as exemplified by the *rapA* promoter (Figure 4A). The position of the canonical IR binding site with respect to the RNAP binding site is suggestive of a type I activation mechanism. Type I activators bind upstream of the -35 region and interact with the flexible α-subunit of RNAP, facilitating recruitment of RNAP to the promoter (37). On the other hand, the position of the DR binding site adjacent to the -35 region is suggestive of a type II activation mechanism. Type II activators bind to a target that often overlaps with the -35 region and typically interact with the sigma-factor by facilitating open complex formation (37). We note that activation of the ComA-regulated *srfAA* promoter is inhibited by the anti-alpha factor SpX (38). This would support a function of ComA as a type I activator. On the other hand, P*_rapA_* (and most other ComA-regulated promoters) require the house-keeping sigma-factor σ^A^, yet interactions of ComA with σ^A^ have not been implied so far.

To investigate how ComA interacts with the transcriptional machinery at the promoter, we conducted EMSAs of P*_rapA_*-DNA with recombinant purified α-subunit from RNAP (Figure 4B) and the sigma-factor σ^A^ from *B. subtilis* (Figure 4C), in the presence and absence of ComA. We found that both the α-subunit and σ^A^ were unable to bind to the promoter in the absence of the transcription factor at the indicated concentrations. However, when ComA was present at concentrations sufficient to fully occupy the promoter, the addition of 0.15 μM of the α-subunit resulted in a super-shift in the EMSA, indicating the formation of a molecular complex with reduced mobility. This suggests that DNA-bound ComA facilitates recruitment of RNAP to the *rapA* promoter by interacting with the α-subunit. Moreover, when the same experiment was conducted with σ^A^, we also observed a super-shift that sets in at 0.1 μM (Figure 4C, right). Together with the results from the promoter activity measurements on constructs containing mutated DR elements (Figure 2), the data are very suggestive of the DR being involved in facilitating a type II activation, while the IR is most likely involved in mediating a type I activation. Promoters in which two transcription factors make independent contacts with RNAP are commonly described as class III promoters (39). Hence ComA could function as a type III transcriptional activator.

### ComA bound to IR and DR motifs interacts differentially with the σ^A^-subunit of RNAP

If a transcription factor is capable of inserting itself into the transcription process in more than one way, and thus effectively acts as a bi-functional transcription factor, alternative binding sites could have evolved to enable it to carry out each separate function. We thus wondered whether the two distinct DNA binding sites might be required to enable proper interactions with RNAP and facilitate type I and type II activation. We therefore studied ternary complex formation by EMSAs using the short synthetic 50 bp PIR and PDR fragments and incubated them with ComA and the α-subunit or σ^A^, respectively.

**Figure 5.**
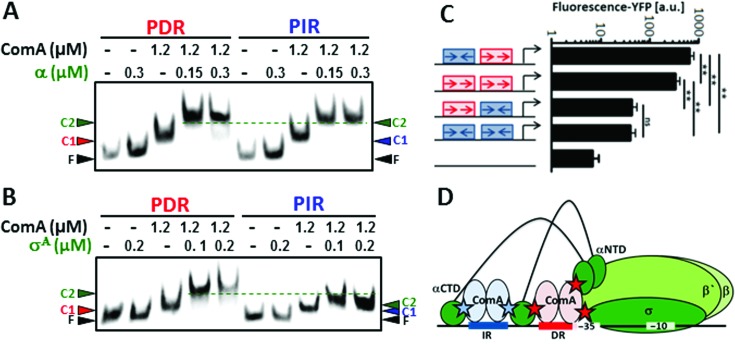
The DR and the IR serve different functions in activating transcription. (**A**) EMSAs of 50 bp fragments containing PDR and PIR-units with ComA and the α-subunit. F: free DNA, C1: Complex with ComA, C2: Ternary complex with the α-subunit. (**B**) Same as in A, but showing interactions with σ^A^. F: free DNA, C1: Complex with ComA, C2: Ternary complex with σ^A^. Note, the DR-bound C2-complex has a lower mobility compared to the IR-bound complex. (**C**) Promoter activities obtained from four synthetic promoter constructs with alternative binding site arrangements PIR-PDR, PDR-PDR, PDR-PIR and PIR-PIR were compared to each other and to the negative control. A DR-site at the class II location is crucial for driving efficient transcription. (**D**) Model of a class III promoter that is activated by ComA.

We first tested the ability of PIR and PDR-bound ComA to recruit the α-subunit (Figure 5A). We found that for both DNA-probes the addition of the α-subunit resulted in a super-shift with respect to the ComA-bound template. Moreover, the shift occurred at the same concentration in both cases and the mobility of the resulting complexes were comparable. This indicates that ComA bound to either the PIR or to the PDR has the same capacity to recruit the α-subunit to the promoter.

Interestingly, when we conducted the same experiment with the σ^A^, we again observed a super-shift for both PIR- and PDR-bound ComA that occured at identical concentrations of σ^A^ (Figure 5B). However, importantly, the mobility of the resulting complexes was different this time. We consistently observed that the molecular complex formed with PIR-bound ComA migrated faster than with PDR-bound ComA. This indicates that the identity of the ComA binding site differentially affects ternary complex formation with σ^A^. One plausible interpretation of the data is that at least one constituent of the resulting complex is present in an altered conformation. Such a conformational change could be important to facilitate steps that occur after recruitment of RNAP to target promoters, such as open complex formation or promoter escape (39).

### The topological properties of the ComA binding site affect transcription *in vivo*

We therefore investigated whether and how the topological identity of the binding sites affects transcription *in vivo*. To this end we designed a reporter construct for an idealized class III promoter with ‘core’ module containing a PIR-PDR binding site arrangement. In addition we designed three synthetic promoters with alternative binding site arrangements, i.e. PDR-PIR, PDR-PDR and PIR-PIR promoters. Figure 5C shows that the topological identity of the binding site has a strong effect on the transcriptional output of the promoter. The ‘ideal’ PIR-PDR construct shows the highest expression level. Importantly, the fluorescence drops more than 10-fold when the PIR element instead of the PDR-element is located proximal to the -35-region in the class II position. Also, in the PDR-PIR construct the activity is very low. Hence, the topological identity of the proximal, i.e. class II binding site, is a strong determinant of the promoter activity. A PDR-bound ComA promotes transcription efficiently while it is severely hampered when ComA is bound to an IR. On the other hand, changing the identity of the distal binding site, i.e. the class I binding site, has a modest effect. The activities of PIR-PIR and PDR-PIR are identical and the activities of PDR-PDR and PIR-PDR differ by less than 2-fold.

Since both idealized binding sites show the same affinity for ComA *in vitro* (Figure 3A), different promoter occupancies are unlikely to be the reason for the striking differences in promoter output. Instead, the data are well explained by our *in vitro* experiments. There is a strong effect of the topology of the ComA binding site on ternary complex formation with σ^A^, suggesting that the two sites might have different capacities to facilitate type II activation (Figure 5B). On the other hand, we could not detect any differences in the interaction pattern with the α-subunit, suggesting that both binding sites when occupied by ComA should function well in RNAP-recruitment (Figure 5A). That the IR nevertheless functions better than the DR as a class I binding site in an *in vivo* context points to some other effect, which may favor this configuration of binding sites.

To summarize, the *in vitro* and *in vivo* data support a model in which the topology of the ComA binding site as IR and DR motifs serve different functions in activating transcription. This is schematically shown in the proposed model summarized in Figure 5D with the distal ComA bound to the IR facilitating RNAP recruitment by interacting with the α-subunit (indicated as a blue star) and a crucial role played by ComA bound at the DR-site in promoting proper interactions with σ^A^ (and potentially making additional contacts with α) thereby facilitating type II activation.

### Regulatory genes in the ICE*Bs*1 element are targets for ComA and regulated in an alternative manner

Although the ComA-mediated quorum response causes genome-wide changes in transcription, relative to the size of the quorum sensing regulon in other bacteria (where more than 100 direct target genes have been identified (12)), the set of genes so far inferred to be directly regulated by ComA is quite small. The current assignment of direct gene targets within the regulon of ComA is mainly based on observations of differential gene expression in microarray experiments and on bioinformatic evidence for the presence of at least one canonical binding site (14). Hence, the putative ability of ComA to bind to an alternative, topologically distinct binding site raises the possibility that a substantial number of genes may exist whose transcription is regulated by ComA exclusively via non-canonical site(s). Such genes would not have been pinpointed as direct targets in earlier analyses. Given the stringent sequence requirements for ComA binding *in vitro*, we reasoned that isolated non-canonical ComA–DNA binding sites in the chromosome of *B. subtilis* should be readily predictable based on sequence alone. We thus scanned the promoter regions of the *B. subtilis* W168 genome for the presence of isolated DRs by imposing the optimal spacer requirement and allowing for a maximum of one mismatched nucleotide in the REs, as suggested by our EMSA experiments. This search identified an isolated putative DR located in the integrative and conjugative element ICE*Bs*1, which can be transferred to various *Bacillus* and *Listeria* species and is a key element in promoting horizontal gene transfer among bacteria (40). The DR is positioned in the intergenic region between *rapI* (which is expressed together with *phrI*) and the divergently transcribed *yddk* gene.

**Figure 6.**
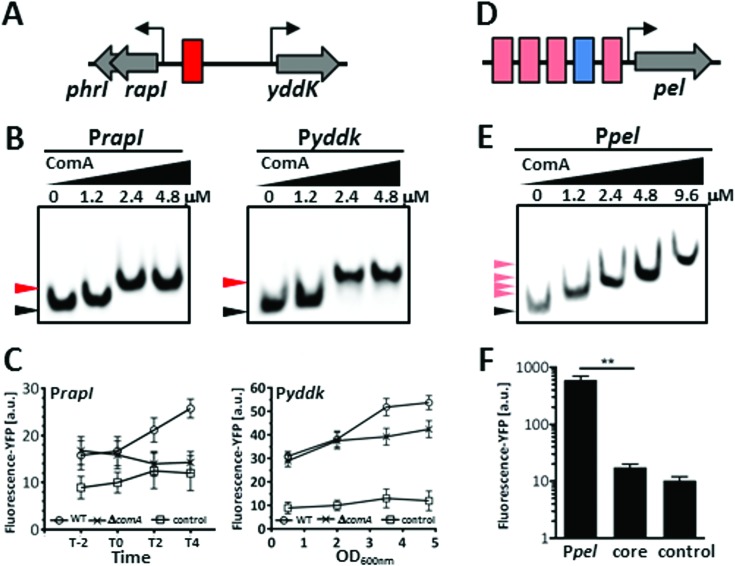
The DR functions in a variety of contexts at different promoters. (**A–C**) Genes in the ICE*Bs*1 element are regulated by binding of ComA to a single non-canonical binding site. (**D–F**) In the *pel* promoter ComA amplifies transcription by binding to a cluster of degenerate non-canonical binding sites. (**A**) A stringent non-canonical binding site (red box) was identified in the intergenic region between the *rapI* and *yddk* genes. (**B**) Results of EMSAs using P*_rapI_* and P*_yddk_* DNAs and ComA. (**C**) Promoter activity measurements of the *rapI* promoter in a wild-type and *ΔcomA* strain cultured in DSM (left). Time is indicated in hours relative to entry into stationary phase (T0). The mean fluorescence level of cells bearing the empty vector is shown as a control. Promoter activity measurements of the *yddk* promoter in a wild-type and *ΔcomA* strain cultured in S7_50_ medium (right). (**D**) Several degenerate DR sites (pale red boxes) form a cluster in the *pel* promoter that is located upstream of an IR–DR core module (blue and red box). (**E**) Results of EMSAs using increasing amounts of ComA and the P*_pel_* promoter, which indicate the formation of various complexes. (**F**) Fluorescence levels obtained from reporter constructs driven by the entire *pel* promoter or the IR–DR core module alone are shown, together with that of the negative control.

In the *rapI* promoter, the DR is located on the minus strand about 30 bp upstream of the putative binding site for RNAP (133 bp upstream of the start of translation). In the case of *yddk*, it lies on the plus strand about 90 bp upstream of the putative RNAP binding site (223 bp upstream of the start of translation) (Figure 6A). EMSAs indeed indicate that ComA is able to bind to the respective promoter regions (Figure 6B). Moreover, we only observed formation of a *single* complex and ComA did not bind to DNA fragments that were devoid of the DR up to 9.6 μM. This indicates that there is only one ComA-binding site present in the respective promoter regions, as was suggested by the sequence analysis. Hence all three genes could be new direct regulatory targets of ComA and regulated in an alternative manner.

While the function of *yddK* is unknown, RapI is a regulatory protein belonging to the Rap-Phr family and stimulates the activity of the ImmA protease, thus permitting derepression and excision of ICE*Bs*1 and thereby promoting its conjugative transfer to recipient cells (41). RapI function is counteracted by the PhrI signaling peptide (40), and the RapI-PhrI signaling system also inhibits sporulation by dephosphorylating a key intermediate in the sporulation phosphorelay (42). We therefore analyzed whether ComA regulates transcription from the DR binding site *in vivo*. To this end, we constructed fluorescence reporter fusions to the *rapI* and the *yddk* promoters and introduced them into the wild-type and a *comA* deletion mutant. For *rapI* promoter activity measurements, strains were grown in DSM. As expected based on RapI's role in regulating sporulation, the *rapI* promoter is induced in a wild-type background, but not in the *comA* mutant, upon entry into stationary phase (Figure 6C, left). Moreover, the expression of the *rapI-phrI* gene cassette has previously been shown to be activated under conditions of high population density (40). Indeed in S7_50_ medium we observed a more gradual induction of the P*_rapI_* with increasing optical density (OD), which was also significantly attenuated in the *comA* mutant (data not shown). A similar behavior was seen for the *yddK* promoter in S7_50_ medium (Figure 6C, right). At low ODs, fluorescence levels in the wild-type and the mutant are indistinguishable. At higher ODs, the activity increases in the wild-type, but the rate of increase is significantly reduced in the *comA* deletion strain. These observations thus support the conclusion that ComA binds directly to the non-canonical binding site in promoters to activate gene expression under conditions of high cell density. Therefore, ComA could play a role in regulating horizontal gene transfer in two ways: by inducing natural "com" petence (to which ComA owes its name (18)) and by promoting conjugation via activation of ICE.

### The architecture of ComA-regulated promoters can be complex

Moreover, at the molecular level, the results for P*_rapI_* and P*_yddK_* imply that ComA can indeed regulate transcription by binding to non-canonical binding sites independently of the presence of an IR. Yet our search for such sequences resulted in only a modest expansion of the set of known direct target genes. The reasons for this may, in part, lie in the exquisite specificity required for binding of ComA to an isolated non-canonical binding site. The chances that such a specific sequence will evolve in promoter regions are low – and its chances of being lost again are high, as a single nucleotide exchange would in many cases be expected to abolish the interaction. On the other hand, many non-canonical and functional binding sites in the IR–DR core module are rather degenerate, and do not comply with the stringent rules suggested by our *in vitro* analysis. We thus searched the upstream promoter regions again with FIMO (43) using the more relaxed criteria depicted in Figure 1B. This resulted in a long list of putative binding site candidates, all showing considerable deviation from the idealized sequence motif.

Interestingly, several additional DR elements were predicted in the *pel* promoter (Figure 6D). This gene codes for pectate lyase C (an enzyme that degrades plant-cell walls), is a known target of ComA. The promoter contains three putative DRs (termed DR1–DR3), clustered upstream of the characteristic ComA core module. The latter is located adjacent to the -35 region and contains one canonical and one non-canonical binding site of the IR and DR-type (termed DR-35). As expected, its promoter sequence binds ComA *in vitro* (Figure 6E). Moreover, with increasing concentrations of ComA additional complexes with lower mobility were observed, indicating the presence of multiple ComA binding sites in the promoter region, as expected. We then assayed for the *in vivo* function of the DR cluster and the core module in the *pel* promoter. To this end, we constructed a truncated version of the P*pel* containing only the ‘core module’, and compared its activity to the intact promoter including the DR cluster. We found that the latter shows a 30-fold increase in fluorescence compared to the truncated core promoter (Figure 6F). Hence ComA binding to the upstream DR cluster greatly amplifies the transcriptional output from the core module.

To summarize, as exemplified by the *rapI, rapA* and *pel* promoters our data show that a non-canonical DR-site can function in a variety of contexts to activate transcription and that the different promoters vary considerable in their degree of complexity.

### Cooperative binding facilitates recognition of more degenerate DR sites

**Figure 7.**
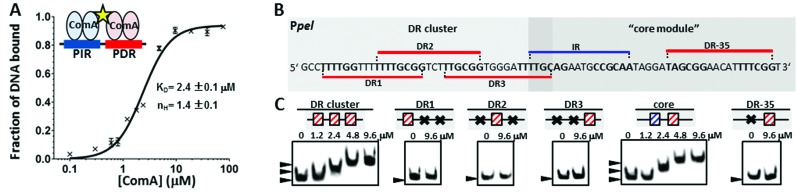
Cooperative interactions facilitate recognition of degenerate binding sites. (**A**) ComA binds to a DNA fragment containing a PIR-PDR core module cooperatively. The binding curve obtained from quantitative EMSAs is shown together with a Hill function fit (black line). Error bars indicate the standard deviation from three independent experiments. (**B**) Sequence of the *pel* promoter which contains four degenerate DR-motifs. DR1-DR3 comprise the upstream ‘DR cluster’ (light grey box), while DR-35 is part of the IR-DR ‘core module’ (dark grey box). (**C**) Results of EMSAs using increasing amounts of ComA and the indicated DNA fragments. Left panel: DR cluster, DR1, 2 and 3. The latter denote mutated ‘DR cluster’ constructs in which only the first (second, etc.) DR-site was left intact. Right panel: IR-DR core and DR-35, in which the IR site was mutated and only DR-35 was left intact. ComA binds to the intact DR cluster and the core module, but is unable to bind to degenerate DR sites individually.

In most cases, promoters contain at least two binding sites, raising the possibility that ComA binding to more complex promoters proceeds cooperatively. To investigate this point, we conducted quantitative EMSAs on DNA fragments containing an idealized PIR-PDR core module. We found that the fraction of DNA bound to ComA increases in a sigmoidal fashion with an apparent Hill coefficient of n_H_ = 1.4 and an apparent K_d_ = 2.4 μM (Figure 7A). This demonstrates that ComA molecules can bind cooperatively to the DNA.

We reasoned that cooperative interactions might also facilitate binding to more degenerate sequences. For example, all four DR elements in the *pel* promoter show considerable degeneracy from the idealized sequence motif (Figure 7B). We thus investigated whether ComA could bind to any of these DR-sites individually, or whether another binding site of the IR or DR-type would be required for binding. To this end, we studied ComA-binding to the DNA-fragment containing the DR-cluster (DR1–DR3) and to three mutant fragments where only one DR binding site was left intact and the nucleotides of the other REs were position-randomized. ComA bound to the intact DR-cluster and with increasing concentration we observed additional complexes with lower mobility. However, ComA did not bind to any of the mutant fragments (Figure 7C, left). Similarly, for a fragment containing the IR-DR core module, ComA was able to bind. However, without the IR-site, ComA could not bind up to 9.6 μM (Figure 7C, right). These findings reinforce the idea that the sequence requirements for binding to an *isolated* DR are rather stringent. However, the presence of several ComA binding sites, regardless of their type (IR or DR), allows for greater flexibility with respect to sequence content. Here, the expected decrease in the binding energy to the degenerate sites might be compensated for by interactions between two or more ComA molecules that bind cooperatively to the promoter.

Given that cooperative interactions promote binding to degenerate sequences, we decided to test three additional gene candidates, *ybaJ, ylbO and ykhA*, which were predicted to contain several degenerate DR, but no IR, elements in their respective promoters. However, none of these was able to bind ComA *in vitro*, nor did the activity of fluorescent fusions to the respective promoters differ between *wt* and *comA* deletion mutant cells in S7_50_ media (data not shown). Therefore, these three genes are unlikely to be direct targets of ComA, at least under our experimental conditions.

## DISCUSSION

Bacterial transcription factors have evolved to act by intervening in the various steps required for transcription, including binding of RNAP, transcription initiation and transcript elongation, or—in the case of promoters regulated by several transcription factors—by modulating the interaction between different transcription factors and/or DNA. The molecular interactions required for a particular mode of regulation impose certain constraints on the position (37,39,44) and orientation (45) of the transcription factor so as to facilitate appropriate contacts with the respective molecular interaction partner(s). Here, we have described a striking example of a transcriptional activator of the quorum response in Gram-positive bacteria that interacts with DNA in more complex ways. We discovered that the quorum sensing master regulator ComA from *B. subtilis* is able to bind to similar DNA motifs organized either as direct or inverted sequence repeats and demonstrated that these sites can function in a variety of different contexts to activate transcription *in vivo*.

In contrast to the case of the quorum sensing master regulator LuxR in *V. harveyi*, where the requirements for binding to alternative DNA-binding motifs correlate with the action of the transcription factor as a repressor or activator, ComA *activates* transcription from both sorts of site. Most ComA-regulated promoters known contain a regulatory core module comprising two binding sites with distinct sequence topologies that are located close to the RNAP binding site. Our *in vitro* and *in vivo* data suggest that the topological properties of the underlying DNA sequence motif are crucial determinants for organizing the transcriptional machinery and facilitate activation of gene expression by recruiting RNAP (type I activation) and by promoting open complex formation (type II activation) as a result of transcription factor binding at each site. We thus propose that the two alternative binding sites at least partially reflect on the versatile interactions with RNAP. Evidence that type I and type II activations may make different demands on the transcription factor with respect to interaction with RNAP comes also from studies on PhoP in *Salmonella enterica*, where different orientations of the binding site with respect to the RNAP binding site correlate with the activation mechanism (45). It thus seems plausible that not only the orientation of the entire binding site—as in the case of PhoP—but also an inherent change in topological properties of a binding site may be required for a transcription factor to interact with the transcriptional machinery in an appropriate way as proposed for ComA. It remains to be established whether the different functions of ComA in activating transcription can be genetically dissected as in the case of quorum sensing master regulator LuxR in *V. harveyi* (12) where the requirements for binding to alternative DNA-binding motifs correlate with the action of the transcription factor as a repressor or activator.

Despite decades of research on how ComA regulates transcription, the alternative binding site had gone unnoticed, probably owing to the considerable level of sequence degeneracy it exhibits and little reason to expect that alternative sites should exist. This degeneracy is in striking contrast to the stringent requirements to facilitate ComA binding to DR sites *in vitro*. From about 20 different DR-type sequences tested here, only 3 were able to bind ComA with lower—albeit still comparable—affinities. No binding was observed for all others which implies that the vast majority of closely related sequences require at least an order of magnitude higher concentrations of ComA for binding, if they bind at all. On the other hand, the presence of two or more ComA sites, (regardless of their identity) facilitates cooperative binding of ComA to the promoter. This suggests that the origin of sequence degeneracy seen in actual promoter sequences is at least in part the result of interactions between transcription factors that stabilize the resulting molecular complexes.

We thus propose that the DNA sequence motif has been shaped substantially by the molecular context, in which the transcription factor operates (and not just by requirements for DNA binding). The DNA sequence evolved and reflects on the constraints imposed by all interactions that the transcription factor faces in the transcription initiation complex (i.e. DNA, RNAP and other transcription factors). Since in most ComA-regulated promoters the DR-site is sandwiched between an IR-site and the -35-site, the DR motif could have been particularly sensitive to influences from interactions on the protein-level. For example, the T at position 1 is highly conserved *in vivo*, while it is dispensable for binding *in vitro*. On the other hand, the actual consensus sequence for the DR elements is in fact asymmetric with a T instead of a G at position 3 in the second RE. Yet, ComA preferred binding to the symmetric site *in vitro*. It is intriguing to speculate that the T1 in RE3 may be important to configure ComA to interact with its partner ComA bound to the IR-site, while T3 in RE4 may be required for interactions with RNAP. In general, how the molecular context in which a specific transcription factor operates shapes the DNA-sequence motif is not well understood. ‘Context’ is thus an emerging theme that is about to change how we analyze the interaction of transcription factors with DNA.

‘Context’ is likely also an important concept for identifying the direct gene targets of multi-functional transcription factors. Here, we described three new ComA-target genes located in the ICEBs1 element, where ComA controls gene expression by acting from a *single* non-canonical binding site. Given the stringent sequence requirements for ComA binding to single sites *in vitro*, we do not expect that many more genes exist which share this type of regulation. However, ComA has the ability to interact with itself which facilitates binding to more degenerate motifs. Moreover, ComA could potentially interact with other transcription factors, which could leave their imprint on the DNA sequence motif. It is thus still possible that many direct targets of ComA remain to be discovered. It is certainly conceivable that ComA exerts its global effects on gene expression via a comparatively modest set of direct targets by exploiting a surprising degree of architectural complexity at the promoter level that evolved in the context of quorum sensing control.

Our results, together with recent findings relating to the TetR-like LuxR homologues in *Vibrio* species, further underline another emerging theme: quorum sensing control systems exhibit characteristics that appear to promote molecular innovation in protein–DNA interactions over the course of evolution. ComA is a member of the tetrahelical HTH subfamily of transcription factors. Since other members of the tetrahelical HTH subfamily are able to bind to DNA as homodimers, monomers (46) or heterodimers (47–49), it is conceivable that this family of transcription factors has evolved the ability to engage in diverse protein–protein interactions, which could have implications for facilitating versatile protein–DNA interactions. Since both full-length ComA (24) and ComAC, a truncated C-terminal version containing the DNA-binding domain (6,26), are known to dimerize readily in solution, we propose that the DR is a binding site for a ComAC *dimer*. Preliminary experiments with ComAC suggest that the truncated protein also binds to a PDR but not to a single RE. Moreover, the stringent sequence requirements for binding to a DR-site, in particular with respect to the spacer-length, further support a dimeric binding model. This distinguishes ComA from other members of the same family, such as NarL from *E. coli* (50), which recognizes both a canonical inverted repeat by binding as a dimer and in addition a single RE probably by binding as a monomer (46).

Binding of a bacterial transcription factor as a homodimer to two topologically distinct DNA binding sites has—to the best of our knowledge—not been reported. However, zinc-finger-based nuclear hormone receptors found in eukaryotes likewise show a remarkable flexibility with respect to recognizing topologically distinct binding sites (51–53). How this is structurally achieved by either class of transcription factors is an open question. Our ComA binding assays with PIR and PDR-sequences yielded remarkably similar results with respect to affinity and the mobility of the resulting complexes (with the important exception of the ternary complex involving σ^A^). Perhaps ComA binding to IR and DR sites proceeds with rather subtle structural changes. The 3D structure of the ComAC dimer in solution has been determined by NMR and was previously used to build a structural model for the interaction of ComAC with an IR sequence (6). We thus wondered whether it was physically possible for this ComAC dimer to bind to a DR sequence while maintaining the experimentally determined dimeric interface. We explored this possibility using flexible protein–DNA docking and tested the stability of the lowest-energy docked conformation of the ComAC-DNA complex with all-atom and explicit water molecular dynamics simulations (54–57),(see Supplementary Material for details). The computational model suggests that ComAC could bind to the DR in a very similar way as to the IR (Supplementary Figures S1 and S2). Although both models still await experimental verification, it is interesting to note that they offer an explanation for the observed response to the nucleotide substitutions in the central GC nucleotides in the second RE (Supplementary Figure S3). A deeper understanding of the molecular properties of the transcription factor and/or the RE to facilitate such topological sequence flexibility should help us to decide whether such promiscuous behavior of a dimeric transcription factor represents a peculiarity of a quorum sensing regulator or whether it could be more prevalent in bacteria.

## Supplementary Material

SUPPLEMENTARY DATA
