# Supplementary material for: The quorum-sensing regulator ComA from Bacillus subtilis activates transcription using topologically distinct DNA motifs
Source: Nucleic Acids Res. 2015 Nov 17;44(5):2160–72. doi: 10.1093/nar/gkv1242 (PMC4797271; doi:10.1093/nar/gkv1242)
Supplement: SUPPLEMENTARY DATA [file supp_44_5_2160__index.html]

The quorum-sensing regulator ComA from Bacillus subtilis activates transcription using topologically distinct DNA motifs — The quorum-sensing regulator ComA from Bacillus subtilis activates transcription using topologically distinct DNA motifs — SUPPLEMENTARY DATA 

# The quorum-sensing regulator ComA from *Bacillus subtilis* activates transcription using topologically distinct DNA motifs

## SUPPLEMENTARY DATA

- SUPPLEMENTARY DATA
- SUPPLEMENTARY DATA
