# Supplementary material for: The quorum-sensing regulator ComA from Bacillus subtilis activates transcription using topologically distinct DNA motifs
Source: Nucleic Acids Res. 2015 Nov 17;44(5):2160–72. doi: 10.1093/nar/gkv1242 (PMC4797271; doi:10.1093/nar/gkv1242)
Supplement: SUPPLEMENTARY DATA [file supp_gkv1242_Supplementary-Material.pdf]

Supplementary Material:

The quorum-sensing regulator ComA from *Bacillus subtilis* activates transcription using topologically distinct DNA motifs

Diana Wolf[1+≠], Valentina Rippa[1+], Juan Carlos Mobarec[2], Patricia Sauer[1], Lorenz Adlung[1], Peter Kolb[2] and Ilka B. Bischofs[1,*]

[1] Center for Molecular Biology (ZMBH) and Center for the Quantitative Analysis of Molecular and Cellular Biosystems (BioQuant), University of Heidelberg, Im Neuenheimer Feld 267, 69120 Heidelberg, Germany

[2] Department of Pharmaceutical Chemistry, Philipps-University Marburg, Marbacher Weg 6, 35032 Marburg, Germany

* **Corresponding Author**

Ilka B. Bischofs
University of Heidelberg
Center for Molecular Biology (ZMBH) and BioQuant
Im Neuenheimer Feld 267, BQ41
69120 Heidelberg, Germany
Tel: +49 6221 54 51365
email: ilka.bischofs@bioquant.uni-heidelberg.de

+: equal contribution
≠ Present address:
Institute of Microbiology, Technical University Dresden, Zellescher Weg 20b, 01217 Dresden, Germany

SUPPLEMENTARY METHODS

Computational modeling of ComAC bound to a perfect direct repeat. A 26-bp DNA sequence (AAACTTTGCGGCCATTTTGCGGTGAT) containing a perfect DR motif (underlined), together with its complementary strand, was modeled using X3DNA (1). The DNA model was built using a geometry analogous to the 3D coordinates of the DNA containing a canonical ComAC binding site (2). We used HADDOCK (3) to model a dimeric ComAC-DR DNA complex. HADDOCK uses Ambiguous Interaction Restraints between so-called ‘active’ residues or nucleotides to decrease the degrees of freedom during exploration of the conformational space. The experimentally validated hexa-nucleotides of the DR were chosen as active nucleotides and the HTH motif of ComAC (determined from the NMR structure (2)) defined the active residues of the protein. Residues and nucleotides surrounding the designated active ones within a 6.5 Å radius were denoted as passive. The best scored clustered structures according to the HADDOCK scoring function were visually inspected, and the single best scored complex was selected. The stability of this molecular complex was tested with molecular dynamics (MD) simulations. All-atom MD simulations were performed with the GROMACS (v 4.5.5) simulation package (4) and the AMBER 99sb force field (5). The docking-derived dimeric ComAC--direct repeat-DNA complex was first energy-minimized with 10^4 steps of the steepest descent algorithm. The complex was solvated in a water box of 94 x 66 x 51 Å and ions were added to ensure electroneutrality of the system. Total system size for a representative run was ~$3*10^4$ atoms, and energy was minimized for 10^4 steps of steepest descent. Positional restraints were applied to the protein and DNA, and the system was run for 12 ps to allow solvent adjustment. Restraints were then removed and three independent production runs of 10, 15, and 25 ns were carried out at 300 °K. The dimeric ComA-DNA complex maintained its organization during the simulations, as evidenced by the root mean square displacement (RMSD) of all atoms with respect to the starting conformation. Mutants were created by replacing bases with the program WITNOTP (Novartis Pharma AG, Basel, Switzerland).

SUPPLEMENTARY FIGURES

Suppplementary Figure S1.

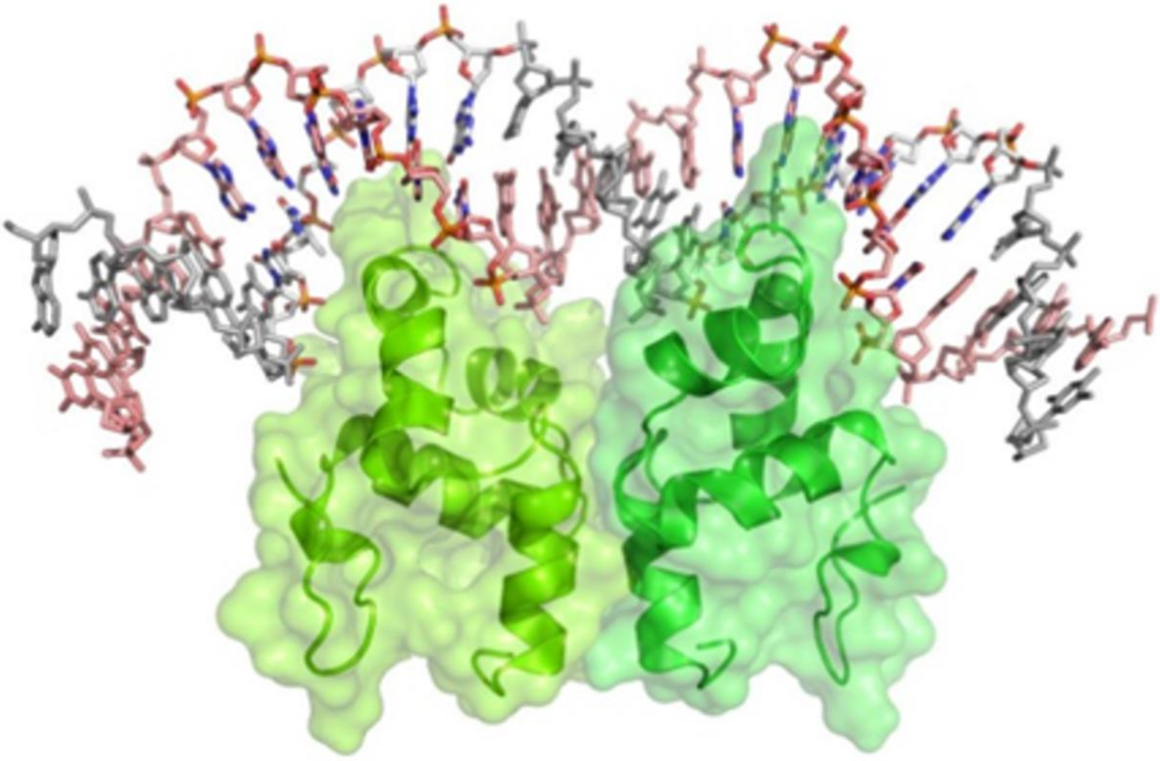

Figure S1. The ComAC dimer docked to DNA containing a perfect direct repeat. Monomers A and B of ComAC are shown in cartoon form and with their accessible surfaces colored in lime and dark green, respectively. The DNA is shown in stick representation with one strand in grey and the other one in salmon. The DR motifs are colored according to the CPK convention, with the carbons colored like their respective strands. The ComA-DR model indicates that the second α-helix of the HTH of each monomer contributes most to DR recognition, binding to the major grooves of the DR-DNA, and interacting mostly with the DNA bases. In addition, several positively charged residues in ComA hold the DNA backbone by establishing electrostatic interactions with the negatively charged phosphate groups. The 3D-coordinates of the docked structure in PDB format can be downloaded as a Supplementary File.

Supplementary Figure S2.

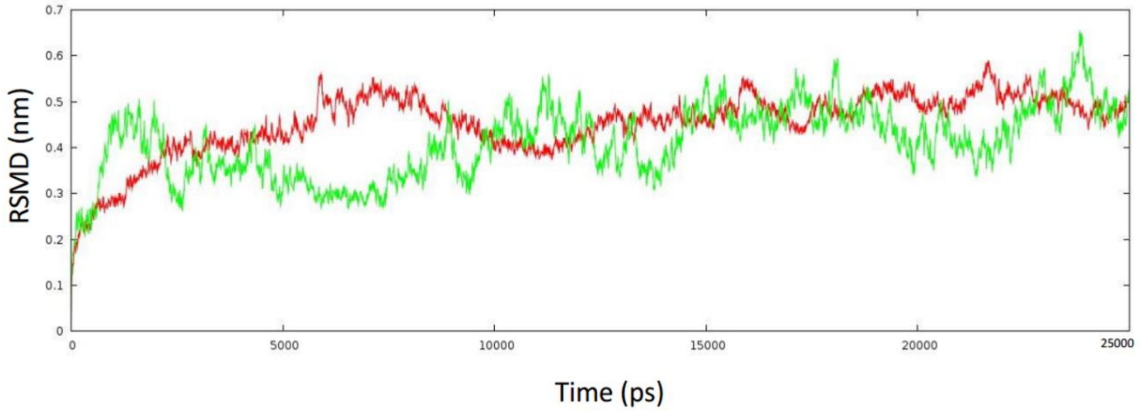

Figure S2. The docked structure is physically stable. The lowest-energy computationally docked conformation of the ComAC-DNA complex was tested for stability with all-atom and explicit water molecular dynamics simulations. The trajectories show the root mean square displacement (RMSD) of the ComAC dimer C-alpha atoms alone (red), and together with the RMSD of the heavy atoms of the DNA (green) with respect to the docked configuration.

Supplementary Figure S3.

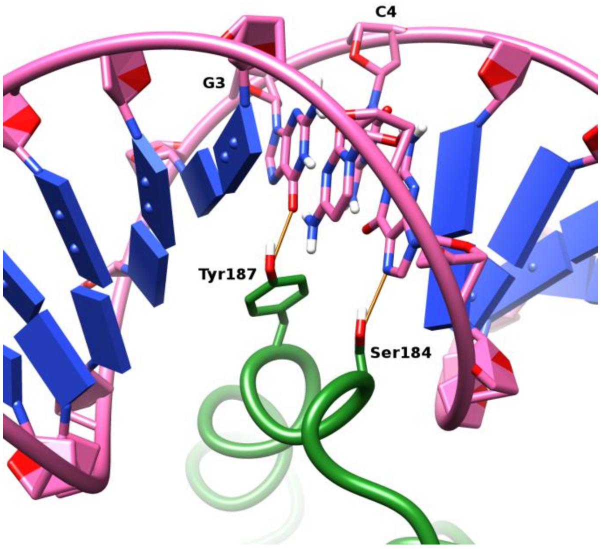

Figure S3. Close-up into the protein-DNA interaction interface of ComAC and a PDR sequence of DNA. The protein is labeled in green, DNA (pink: carbons, blue bases). Hydrogen bonds are marked by orange lines and residues interacting with the central GC bases are labeled. In the PDR the G at position 3 of the second RE makes a hydrogen bond with Tyr187. When the G is replaced by T, the hydrogen bond is maintained leading to binding in both cases. In contrast, the C4A mutation abolishes binding, as the G on the opposite strand interacts with Ser184 via a hydrogen bond. A T at this position points its methyl group towards the serine, thereby breaking this interaction. At the same time, a more detailed inspection of the model of ComAC interacting with the canonical DNA sequence (from Ref. (2)) yields similar structural explanations for the observed binding events in the experimental section. For G3T of the IR, the methyl group of T creates a more hydrophobic environment, which is unfavorable given the close proximity of Arg183. C4A is a neutral mutation, as both bases can maintain similar polar interactions with the interacting protein residue Arg183.

SUPPLEMENTARY FILES

File 1. Coordinates of ComAC docked to the perfect direct repeat DNA in pdb-format.

SUPPLEMENTARY TABLES

Table 1. *Bacillus subtilis* strains used in this study. RE: recognition element; ↓ mutation away from the wild-type sequence; ↑ mutation towards the idealized sequence; PIR: perfect inverted repeat; PDR: perfect direct repeat.

Strains	Genotype	Reference or source
**W168**	*trpC2*	Oskar Kuipers (1A700)
**JH642**	*pheA1*, *trpC2*, *citS642*	BGSC (1A96)
**BIB336**	JH642 *bglS*::piYFPbglS (empty vector control)	This study
**BIB339**	W168 *bglS*::piYFPbglS (empty vector control)	This study
**BIB372**	JH642 pIB69 (pLK-P*spac-comK*)	This study
**BIB396**	JH642 *ΔcomA,* pIB69 (pLK-P*spac-comK*)	This study
**BIB380**	JH642 *bglS*::pDW37 (P_{srfAA}-Box1+2-RE4↓-iYFP)	This study
**BIB477**	JH642 *bglS*::pDW40 (P_{srfAA}-Box1+2-iYFP)	This study
**BIB503**	JH642 *bglS*::pDW60 (P_{rapA}-iYFP)	This study
**BIB507**	JH642 *bglS*::pDW53 (P_{srfAA}-Box1+2-RE3↓+RE4↓-iYFP)	This study
**BIB513**	JH642 *bglS::*pDW58 (P_{rapC}-iYFP)	This study
**BIB515**	JH642 *bglS::*pDW61 (P_{rapA}-RE4↓-iYFP)	This study
**BIB519**	JH642 *bglS::*pDW63 (P_{rapA}-RE3↓+RE4↓-iYFP)	This study
**BIB527**	JH642 *bglS::*pDW72 (P_{lutP}-RE3↓+RE4↓-iYFP)	This study
**BIB573**	JH642 *bglS::*pDW73 (P_{rapC}-RE4↓-iYFP)	This study
**BIB575**	JH642 *bglS::*pDW74 (P_{rapC}- RE3↓+RE4↓-iYFP)	This study
**BIB577**	JH642 *bglS::*pDW75 (P_{rapC}-RE4↑-iYFP)	This study
**BIB581**	JH642 *bglS::*pDW68 (P_{lutP}-iYFP)	This study
**BIB734**	W168 *bglS*::pDW99 (PDR-PDR)	This study
**BIB736**	W168 *bglS*::pDW100 (PDR-PIR)	This study
**BIB766**	W168 *bglS*::pDW97 (PIR-PIR)	This study
**BIB768**	W168 *bglS*::pDW98 (PIR-PDR)	This study
**BIB830**	W168 *bglS::*pDW124 (P_{pel}-IR+DR-iYFP)	This study
**BIB897**	W168 *bglS*::pDW67 (P_{pel}-iYFP)	This study
**BIB1068**	JH642 *bglS*::pVR1(P_{rapI}-iYFP)	This study
**BIB1072**	JH642 *ΔcomA, bglS*::pVR1(P_{rapI}-iYFP)	This study
**BIB1070**	JH642 *bglS*::pVR2(P_{yddk}-iYFP)	This study
**BIB1074**	JH642 *ΔcomA, bglS*::pVR2(P_{yddk}-iYFP)	This study

Table 2. Plasmids used in this study. *Bla*: ampicillin; *kan*: kanamycin; *erm*: erythromycin; *spc*: spectinomycin; LIC: ligation independent cloning; RE: recognition element; IR: inverted repeat; DR: direct repeat. ↓ mutation away from the wild type sequence; ↑ mutation towards the idealized sequence; PIR: perfect inverted repeat; PDR: perfect direct repeat.

Plasmid	Description	Primer used for cloning	Reference or source
**piYFPbglS**	*bglS*'…'*bglS*,*bla*,*kan*,iYFP,ColE1,LIC		Ref. (6)
**pGFPbglS**	*bglS*'…'*bglS*,*bla*,*kan*,GFP,ColE1,LIC		Ref. (28)
**pBAD18**	pBR322 ori, *araC*, P_{BAD}, *bla, rrnB*T		Ref. (7)
**pMAD**	pBR322 ori, pE194-Ts ori, P*clpB*, *bgaB*, *bla, erm*		Ref. (8)
**pLK**	*bla*, *spc*, P*spac*, *comK*, P*divIVA*, *lacZ*, *lacI*, pLS20 replicon		Ref. (9)
**pDW6**	pMAD-*comA*-up/down	DW41/DW8+DW42/DW9	This study
**pDW7**	pBAD18-His6-*com*A	DW1/DW2	This study
**pDW37**	P_{srfAA}-Box1+2-RE4↓-iYFP	DW87/ST15+DW86/LA16	This study
**pDW40**	P_{srfAA}-Box1+2 –iYFP	ST15/LA16	This study
**pDW53**	P_{srfAA}-Box1+2-RE3↓+RE4↓-iYFP	DW91/ST15+DW90/LA16	This study
**pDW54**	P_{srfAA}-PIR-PIR- GFP	DW78/ST11+DW77/ST11	This study
**pDW55**	P_{srfAA}-PIR-PDR- GFP	DW76/ST11+DW77/ST11	This study
**pDW56**	P_{srfAA}-PDR-PDR- GFP	DW76/DW11+DW79/ST11	This study
**pDW57**	P_{srfAA}-PDR-PIR- GFP	DW78/ST11+DW79/ST11	This study
**pDW58**	P_{rapC}-iYFP	DW44/DW45	This study
**pDW60**	P_{rapA}-iYFP	DW55/DW56	This study
**pDW61**	P_{rapA}-RE4↓-iYFP	DW106/DW56	This study
**pDW63**	P_{rapA}-RE3↓+RE4↓-iYFP	DW107/DW56	This study
**pDW67**	P_{pel}-iYFP	DW93/DW94	This study
**pDW68**	P_{lutP}-iYFP	DW101/DW102	This study
**pDW72**	P_{lutP}-RE3↓+RE4↓-iYFP	DW108/DW102	This study
**pDW73**	P_{rapC}-RE4↓-iYFP	DW122/DW45	This study
**pDW74**	P_{rapC}-RE3↓+RE4↓-iYFP	DW123/DW45	This study
**pDW75**	P_{rapC}-RE4↑-iYFP	DW124/DW45	This study
**pDW97**	P_{srfAA}-PIR-PIR- iYFP	DW77/ST11(template pDW54)	This study
**pDW98**	P_{srfAA}-PIR-PDR- iYFP	DW77/ST11(template pDW55)	This study
**pDW99**	P_{srfAA}-PDR-PDR- iYFP	DW79/ST11(template pDW56)	This study
**pDW100**	P_{srfAA}-PDR-PIR- iYFP	DW79/ST11(template pDW57)	This study
**pDW124**	P_{pel}-IR+DR-iYFP	DW195/DW94	This study
**pVR1**	P_{rapI}-iYFP	FwP*rapI*(LIC)/RvP*rapI*(LIC)	This study
**pVR2**	P_{yddk}-iYFP	FwP*yddk*(LIC)/RvP*yddk*(LIC)	This study

Table 3. Oligonucleotides used in this study. LIC sites are in capitals; nucleotide exchanges are in bold capitals.

Primer name and purpose	Sequence
**LIC cloning of promoter fusions in pYFPbglS**	
**LA16 P$_{srfAA}$-LIC rev**	GTTCCTCCTTCCCACCgcatccgttaaagggtaaaaa
**ST11 P$_{srfAA}$ rev trunc LIC Bisi**	GTTCCTCCTTCCCACCttttatctttctaccgttcag
**ST15 P$_{srfAA}$ fwd full length LIC**	CCGCGGGCTTTCCCAGCgtttagtggaaatgattgcg
**DW44 P$_{rapC}$-LIC-fwd**	CCGCGGGCTTTCCCAGCgcacaccgcttcggtttattgcgtg
**DW45 P$_{rapC}$-LIC-rev**	GTTCCTCCTTCCCACCccgaccgctgaagaaggaattac
**DW55 P$_{rapA}$-LIC-fwd**	CCGCGGGCTTTCCCAGCgttgcggttagccgaaattcgac
**DW56 P$_{rapA}$-LIC-rev**	GTTCCTCCTTCCCACCgtctgcttcatcctcaattaatc
**DW76 P$_{srfAA}$-PIR-PDR fwd1**	gaaacttt**G**c**GG**ccatttt**G**cggtgataaaaac
**DW77 P$_{srfAA}$-PIR-PDR fwd2 (LIC)**	gaaacttt**G**c**GG**ccatttt**G**cggtgataaaaac
**DW78 P$_{srfAA}$-PDR-PIR fwd1**	gaaacttt**G**cggcatcccgca**A**tgataaaaacatttttttc
**DW79 P$_{srfAA}$-PDR-PIR fwd2 (LIC)**	CCGCGGGCTTTCCCAGCgaaacttt**G**c**GG**ccatttt**G**cgggaaactttgcgg
**DW86 P$_{srfAA}$ RE3wt-RE4no fwd**	gaaacttttcacccatt**CGCGTA**tgataaaaacattt
**DW87 P$_{srfAA}$ RE3wt-RE4no rev**	ca**TACGCG**aatgggtgaaaagtttc
**DW90 P$_{srfAA}$ RE3+4no fwd**	gaaact**GCGATG**ccatt**GCGATG**tgataaaaac
**DW91 P$_{srfAA}$ RE3+4no rev**	gtttttatca**CATCGC**aatgg**CATCGC**agtttc
**DW93 P$_{pel}$ fwd (LIC)**	CCGCGGGCTTTCCCAGCgggcataaagcaaggaaaaaacc
**DW94 P$_{pel}$ rev (LIC)**	GTTCCTCCTTCCCACCcaaagccgtagctaacatcac
**DW101 P$_{lutP}$ fwd (LIC)**	CCGCGGGCTTTCCCAGCggctgtttacgatctattatac
**DW102 P$_{lutP}$ rev (LIC)**	GTTCCTCCTTCCCACCcctataggcgtatatgcctgtg
**DW106 P$_{rapA}$ RE3wt-RE4no fwd (LIC)**	CCGCGGGCTTTCCCAGCgttgcggttagccgaaattcgacaattgcggttatt**GAT**cg**C**tcttctttttc
**DW107 P$_{rapA}$ RE3+RE4no fwd (LIC)**	CCGCGGGCTTTCCCAGCgttgcggttagccgaaattcgacaa**GC**g**AT**gttatt**GAT**cg**C**tcttctttttc
**DW108 P$_{lutP}$ RE3+RE4no fwd (LIC)**	CCGCGGGCTTTCCCAGCgttttgtgggccgccaaaagattttgactatt**CG**gtcaaaaag**G**ctt**C**tactttg
**DW122 P$_{rapC}$ RE3wt-RE4no fwd (LIC)**	CCGCGGGCTTTCCCAGCggtttattgcgtgctcccgaaaacaaagattgcgtgtttt**A**c**C**g**C**ttgggacg
**DW123 P$_{rapC}$ noRE3+4 fwd (LIC)**	CCGCGGGCTTTCCCAGCggtttattgcgtgctcccgaaaacaaaga**GC**g**ATG**gtttt**A**c**C**g**C**ttgggacg
**DW124 P$_{rapC}$ full-RE4cons. fwd (LIC)**	CCGCGGGCTTTCCCAGCggtttattgcgtgctcccgaaaacaaagattgcgtgttttt**T**g**C**g**G**tgggacg
**Fw P*rapI* (LIC)**	CCGCGGGCTTTCCCAGCgctatgaccgaaaaaaaaaccgcaa
**Rv P*rapI* (LIC)**	GTTCCTCCTTCCCACCgtgcgaatttttacattaattg
**Fw P*yddk* (LIC)**	CCGCGGGCTTTCCCAGCttcctttgcggttttttttcgg
**Rv P*yddk* (LIC)**	GTTCCTCCTTCCCACCtccattgtttgtaaacatttc
**DW195 P$_{pel}$ trunc fwd (LIC)**	CCGCGGGCTTTCCCAGCttgcagaatgccgcaataggat
**Primers for verifying fluroescent promoter constructs in pYFPbglS**	
**LA21 piYFP-check rev**	tgaatccatagtagttcctcc
**LA22 piYFP-check fwd**	agtgaatttaggaggcttactt
**LA38 bglS_fup_LA-rev**	cggctctacaaagacgaatttg
**ComA-His6 overexpression in pBAD18**	
**DW1 *comA*-His6-fwd (KpnI)**	gcttagtgggtaccaaggagatatacatatgcatcaccatcaccatcacaaaaagatactagtgattga
**DW2 *comA*-rev (SphI)**	tgctaccagcatgcttaaagtacaccgtctga

**α-His6 overexpression in pBAD18**	
**VR98 *rpoA*-His6-fwd KpnI**<br>**VR99 *rpoA* Rev SphI**	ccatggtaccaaggagatatacatatgcatcaccatcaccatcacatcgagattgaaaaaccaaaaatcg<br>ccatgcatgctcaatcgtctttgcgaagtccgagtcc

**σ-His6 overexpression in pBAD18**	
**VR100**<br>**VR101**	ccatggtaccaaggagatatacatatgcatcaccatcaccatcacgctgataaacaaacccacgagac<br>ccatgcatgcttattcaaggaaatctttcaaacgtttacttctgc

***comA* clean deletion using pMAD**	
**DW41 *comA* up fwd (BamHI)**	agccggatccgaggttactcctgatcacaaagtg
**DW8 *comA*-up rev**	catatccatctctacaccccccaaccttttactcactcttttccatt
**DW9 *comA*-do fwd**	gttggggggtgtagagatggatatg
**DW42 *comA* do rev (EcoRI)**	ccatgaattccaatcggcatcaatctgatcgc

**EMSA substrates and primers for generating EMSA substrates**	
**Fw PDR**	[Btn]aaactggaatcgaaactttgcggccattttgcggtgataaaaacattttt
**Rv PDR**	aaaaatgtttttatcaccgcaaaatggccgcaaagtttcgattccagttt
**Fw PDR G3→T**	[Btn]aaactggaatcgaaactttgcggccatttttcggtgataaaaacattttt
**Rv PDR G3→T**	aaaaatgtttttatcaccgaaaaatggccgcaaagtttcgattccagttt
**Fw PDR C4→A**	[Btn]aaactggaatcgaaactttgcggccattttgaggtgataaaaacattttt
**Rv PDR C4→A**	aaaaatgtttttatcacctcaaaatggccgcaaagtttcgattccagttt
**Fw NC**	[Btn]aaactggaatcgaaactgcggttccattcgtggttgataaaaacattttt
**Rv NC**	aaaaatgtttttatcaaccacgaatggaaccgcagtttcgattccagttt
**Fw PDR n1**	[Btn]aaactggaatcgaaactttgcggcttgcggtgataaaaacattttt
**Rv PDR n1**	aaaaatgtttttatcaccgcaagccgcaaagtttcgattccagttt
**Fw PDR n2**	[Btn]aaactggaatcgaaactttgcggccttgcggtgataaaaacattttt
**Rv PDR n2**	aaaaatgtttttatcaccgcaaggccgcaaagtttcgattccagttt
**Fw PDR n3**	[Btn]aaactggaatcgaaactttgcggccattgcggtgataaaaacattttt
**Rv PDR n3**	aaaaatgtttttatcaccgcaatggccgcaaagtttcgattccagttt
**Fw PDR n4**	[Btn]aaactggaatcgaaactttgcggccatttgcggtgataaaaacattttt
**Rv PDR n4**	aaaaatgtttttatcaccgcaaatggccgcaaagtttcgattccagttt
**Fw PDR n7**	[Btn]aaactggaatcgaaactttgcggccattacttgcggtgataaaaacattttt
**Rv PDR n7**	aaaaatgtttttatcaccgcaagtaatggccgcaaagtttcgattccagttt
**Fw PDR n9**	[Btn]aaactggaatcgaaactttgcggccattaccattgcggtgataaaaacattttt
**Rv PDR n9**	aaaaatgtttttatcaccgcaatggtaatggccgcaaagtttcgattccagttt
**Fw PDR n10**	[Btn]aaactggaatcgaaactttgcggccattaccatttgcggtgataaaaacattttt
**Rv PDR n10**	aaaaatgtttttatcaccgcaaatggtaatggccgcaaagtttcgattccagttt
**Fw PDR n15**	[Btn]aaactggaatcgaaactttgcggccattaccatctactttgcggtgataaaaacattttt
**Rv PDR n15**	aaaaatgtttttatcaccgcaaagtagatggtaatggccgcaaagtttcgattccagttt
**Fw PIR**	[Btn]atgtttagtggaaatgattgcggcatcccgcaaaaaatattgctgtaaat
**Rv PIR**	atttacagcaatattttttgcgggatgccgcaatcatttccactaaacat
**Fw PIR G3→T**	[Btn]atgtttagtggaaatgattgcggcatccctcaaaaaatattgctgtaaat
**Rv PIR G3→T**	atttacagcaatattttttgagggatgccgcaatcatttccactaaacat
**Fw PIR C4→A**	[Btn]atgtttagtggaaatgattgcggcatcccgaaaaaaatattgctgtaaat
**Rv PIR C4→A**	atttacagcaatatttttttcgggatgccgcaatcatttccactaaacat
**Fw PIR n1**	[Btn]atgtttagtggaaatgattgcggcccgcaaaaaatattgctgtaaat
**Rv PIR n1**	atttacagcaatattttttgcgggccgcaatcatttccactaaacat
**Fw PIR n2**	[Btn]atgtttagtggaaatgattgcggcaccgcaaaaaatattgctgtaaat

**Rv PIR n2**	atttacagcaatatttttgcggtgccgcaatcatttccactaaacat
**Fw PIR n3**	[Btn]atgtttagtggaaatgattgcggcatccgcaaaaaatattgctgtaaat
**Rv PIR n3**	atttacagcaatatttttgcggatgccgcaatcatttccactaaacat
**Fw PIR n5**	[Btn]atgtttagtggaaatgattgcggcatctccgcaaaaaatattgctgtaaat
**Rv PIR n5**	atttacagcaatatttttgcggagatgccgcaatcatttccactaaacat
**Fw PIR n6**	[Btn]atgtttagtggaaatgattgcggcatctcccgcaaaaaatattgctgtaaat
**Rv PIR n6**	atttacagcaatatttttgcgggagatgccgcaatcatttccactaaacat
**Fw PIR n8**	[Btn]atgtttagtggaaatgattgcggcatctccaccgcaaaaaatattgctgtaaat
**Rv PIR n8**	atttacagcaatatttttgcggtggagatgccgcaatcatttccactaaacat
**Fw PIR n10**	[Btn]atgtttagtggaaatgattgcggcatctccacaccgcaaaaaatattgctgtaaat
**Rv PIR n10**	atttacagcaatatttttgcggtgtggagatgccgcaatcatttccactaaacat
**Fw PIR n14**	[Btn]atgtttagtggaaatgattgcggcatctccacataccccgcaaaaaatattgctgtaaat
**Rv PIR n14**	atttacagcaatatttttgcggggtatgtggagatgccgcaatcatttccactaaacat
**Fw RE**	[Btn]aaactggaatcgaaactttgcggccattgcggtttgataaaaacattttt
**Rv RE**	aaaaatgtttttatcaaaccgcaatggccgcaaagtttcgattccagttt
**DW145 PDR nt ex1 fwd (LIC)**	CCGCGGGCTTTCCCAGCgaaact**C**tgcggccattttgcgg
**Fw PDR nt ex2**	[Btn]aaactggaatcgaaactt**C**gcggccattttgcggtgataaaaacattttt
**Rv PDR nt ex2**	aaaaatgtttttatcaccgcaaaatggccgcgaagtttcgattccagttt
**DW159 PDR nt ex3 fwd (LIC)**	CCGCGGGCTTTCCCAGCgaaacttt**A**cggccattttgcgg
**Fw PDR nt ex4**	[Btn]aaactggaatcgaaactttg**T**ggccattttgcggtgataaaaacattttt
**Rv PDR nt ex4**	aaaaatgtttttatcaccgcaaaatggccacaaagtttcgattccagttt
**DW161 PDR nt ex5 fwd (LIC)**	CCGCGGGCTTTCCCAGCgaaactttgc**A**gccattttgcgg
**Fw PDR nt ex6**	[Btn]aaactggaatcgaaactttgcg**A**ccattttgcggtgataaaaacattttt
**Rv PDR nt ex6**	aaaaatgtttttatcaccgcaaaatggtcgcaaagtttcgattccagttt
**DW191PDR nt ex-spacer fwd(LIC)**	CCGCGGGCTTTCCCAGCgaaactttgcggcc**G**ttttgcgg
**Fw PDR nt ex7**	[Btn]aaactggaatcgaaactttgcggccatt**C**tgcggtgataaaaacattttt
**Rv PDR nt ex7**	aaaaatgtttttatcaccgcagaatggccgcaaagtttcgattccagttt
**Fw PDR nt ex8**	[Btn]aaactggaatcgaaactttgcggccattt**C**gcggtgataaaaacattttt
**Rv PDR nt ex8**	aaaaatgtttttatcaccgcgaaatggccgcaaagtttcgattccagttt
**Fw PDR nt ex9**	[Btn]aaactggaatcgaaactttgcggccatttt**A**cggtgataaaaacattttt
**Rv PDR nt ex9**	aaaaatgtttttatcaccgtaaaatggccgcaaagtttcgattccagttt
**Fw PDR nt ex10**	[Btn]aaactggaatcgaaactttgcggccattttg**T**ggtgataaaaacattttt
**Rv PDR nt ex10**	aaaaatgtttttatcaccacaaaatggccgcaaagtttcgattccagttt
**DW164 PDR nt ex11 fwd (LIC)**	CCGCGGGCTTTCCCAGCgaaactttgcggccattttgc**A**gtgataaaaac
**DW163 PDR nt ex12 fwd (LIC)**	CCGCGGGCTTTCCCAGCgaaactttgcggccattttgcg**A**tgataaaaac
**ST11 P*srfAA* rev trunc (LIC)**	GTTCCTCCTTCCCACCttttatctttctaccgttcag
**Fw *rapA***	[Btn]cgtgctttttttgttgcggttagc
**Rv *rapA***	ttaatcccccccttttgaattaccc
**Fw P*rapI***	[Btn]gttactgtcaagtcgcaaaag
**Rv P*rapI***	gaaaattcccccatccttac
**Fw NC*rapI***	[Btn]gttactgtcaagtcgcaaaagaattgctatgaaaaccgaaaaaaaaccgcaggaat
**Rv NC*rapI***	gaaaattcccccatccttacataaaattgtgcg
**Fw P*yddk***	[Btn]gcaaaaaggtaccttatatctattcc
**Rv P*yddk***	tagatttcctccgacctaatttaatc
**Fw NC*yddk***	[Btn]gcaaaaaggtaccttatatctattcctgcggttttttttcggttttcatag
**Rv NC*yddk***	tagatttcctccgacctaatttaatccattgtttg
**Fw P*pel***	[Btn]aaaaccaaaaggccaatgtcgg
**Rv P*pel***	cacaaaaaatataatagcaaattgaggg
**Fw cluster DR**	[Btn]cggccttttggtttttttgcggtctttgcggtgggattttgcagaat
**Rv cluster DR**	attctgcaaaatcccaccgcaaagaccgcaaaaaaaccaaaaggccg
**Fw core**	[Btn]ggattttgcagaatgccgcaataggatagcggaacattttcggttctg
**Rv core**	cagaaccgaaaatgttccgctatcctattgcggcattctgcaaaatcc
**Fw DR1**	[Btn]cggccttttggtttttttgcggtctggctgttgggagtctttagaat
**Rv DR1**	attctaaagactcccaacagccagaccgcaaaaaaaccaaaaggccg
**Fw DR2**	[Btn]cggccggttttttttttttgcggtctttgcggtgggagtctttagaat
**Rv DR2**	attctaaagactcccaccgcaaagaccgcaaaaaaaaaaaaccggccg
**Fw DR3**	[Btn]cggccggtttttttttggctgttctttgcggtgggattttgcagaat
**Rv DR3**	attctgcaaaatcccaccgcaaagaacagccaaaaaaaaaaccggccg
**Fw DR-35**	[Btn]aatgaaccgctaggatagcggaacattttcggttctgaatgtccctc
**Rv DR-35**	gagggacattcagaaccgaaaatgttccgctatcctagcggttcatt

Table 4. Relevant sequences of mutant and synthetic promoter constructs

**P*srfAA* RE3+RE4 wild type**	TTTCACccattTTTCGG
**P*srfAA* no RE3+RE4**	GCGATGccattGCGATG
**P*srfAA* no RE4**	TTTCACccattCGCGTA
**P*rapA* RE3+RE4 wild type**	TTGCGGttattTTGCGT
**P*rapA* no RE3+RE4**	GCGATGttattGATCGC
**P*rapA* no RE4**	TTGCGGttattGATCGC
**P*lutP* RE3+RE4 wild type**	TTGTCAaaaagTCTTGT
**P*lutP* no RE3+RE4**	CGGTCAaaaagGCTTCT
**P*rapC* RE3+RE4 wild type**	TTGCGTgttttTCGGGT
**P*rapC* no RE3+RE4**	GCGATGgttttACCGCT
**P*rapC* no RE4**	TTGCGTgttttACCGCT
**P*rapC* perfect RE4**	TTGCGTgttttTTGCGG
**PIR-PDR**	TTGCGGcatcCCGCAAgaaactTTGCGGccattTTGCGG
**PDR-PDR**	TTGCGGccattTTGCGGgaaactTTGCGGccattTTGCGG
**PDR-PIR**	TTGCGGccattTTGCGGgaaactTTGCGGcatcCCGCAA
**PIR-PIR**	TTGCGGcatcCCGCAAgaaactTTGCGGcatcCCGCAA

SUPPLEMENTARY REFERENCES

1. Zheng,G., Lu,X.J. and Olson,W.K. (2009) Web 3DNA - A web server for the analysis, reconstruction, and visualization of three-dimensional nucleic-acid structures. *Nucleic Acids Res.*, **37**, 240–246.

2. Hobbs,C. A., Bobay,B.G., Thompson,R.J., Perego,M. and Cavanagh,J. (2010) NMR solution structure and DNA-binding model of the DNA-binding domain of competence protein A. *J. Mol. Biol.*, **398**, 248–63.

3. Dominguez,C., Boelens,R. and Bonvin,A.M.J.J. (2003) HADDOCK: A protein-protein docking approach based on biochemical or biophysical information. *J. Am. Chem. Soc.*, **125**, 1731–1737.

4. Pronk,S., Páll,S., Schulz,R., Larsson,P., Bjelkmar,P., Apostolov,R., Shirts,M.R., Smith,J.C., Kasson,P.M., Van Der Spoel,D., *et al.* (2013) GROMACS 4.5: A high-throughput and highly parallel open source molecular simulation toolkit. *Bioinformatics*, **29**, 845–854.

5. Hornak,V., Abel,R., Okur,A., Bentley,S., Adrian,R. and Simmerling C. (2008) Comparison of Multiple Amber Force Fields and Development of Improved Protein Backbone Parameters. *Proteins*, **65**, 721–725.

6. Bisicchia,P., Botella,E. and Devine,K.M. (2010) Suite of novel vectors for ectopic insertion of GFP, CFP and IYFP transcriptional fusions in single copy at the amyE and bglS loci in Bacillus subtilis. *Plasmid*, **64**, 143–9.

7. Guzman,L.M., Belin,D., Carson,M.J. and Beckwith,J. (1995) Tight regulation, modulation, and high-level expression by vectors containing the arabinose. These include : Tight Regulation, Modulation, and High-Level Expression by Vectors Containing the Arabinose PBAD Promoter. **177**, 4121–4130.

8. Arnaud,M., Chastanet,A. and De,M. (2004) New Vector for Efficient Allelic Replacement in Naturally Gram-Positive Bacteria. *Appl Environmental Microbiol*, **70**, 6887–6891.

9. Nijland,R., Burgess,J.G., Errington,J. and Veening,J.W. (2010) Transformation of environmental Bacillus subtilis isolates by transiently inducing genetic competence. *PLoS One*, **5**, e9724.
